# A tale of two agricultural revolutions: crop introductions in the long 1st millennium ce southern Levant

**DOI:** 10.1007/s00334-025-01060-9

**Published:** 2025-10-10

**Authors:** Annette M. Hansen, Frits Heinrich, Dafna Langgut, Ehud Weiss, Daniel Fuks

**Affiliations:** 1 Groningen Institute of Archaeology, University of Groningen, Groningen, The Netherlands; 2 Research Group Interdisciplinary Historical Food Studies (FOST), Department of History, Archaeology, Arts, Philosophy and Ethics, Vrije Universiteit Brussel, Brussels, Belgium; 3 Research Group Industrial Microbiology and Food Biotechnology (IMDO), Department of Bioengineering Sciences, Vrije Universiteit Brussel, Brussels, Belgium; 4 The Laboratory of Archaeobotany and Ancient Environments, Institute of Archaeology, The Steinhardt Museum of Natural History, Tel Aviv University, Tel Aviv, Israel; 5 Archaeobotany Lab, Department of Land of Israel Studies and Archaeology, Bar-Ilan University, Martin, Ramat Gan, Szusz Israel; 6 Archaeobotany and Environmental Archaeology Lab, Department of Archaeology, Ben-Gurion University of the Negev, Beersheba, Israel

**Keywords:** Agricultural history, Roman Agricultural Diffusion, Islamic Green Revolution, Archaeobotany, Crop history

## Abstract

This paper aims to provide a robust regional synthesis of crop diffusion in the southern Levant, with the objective of ascertaining the significance of 1st millennium ce introductions, including those associated with Roman Agricultural Diffusion (RAD) and the Islamic Green Revolution (IGR). It does so by integrating published archaeobotanical data and written evidence from multiple periods to identify the earliest attestations of crop taxa. We present the earliest evidence for 80 crops in the region from the Neolithic until the Late Medieval period, focusing on ‘the long 1st millennium ce’. We will not only consider first finds, but also, where possible, the qualitative evidence for adoption and entrenchment of these crop plants in this region. Our findings indicate that RAD crops are numerically more significant than IGR crops, although the difference is greater in the archaeobotanical record compared with the textual evidence. Nearly half of the surveyed crops for which we have reliable data were introduced to the southern Levant in the 1st millennium ce, indicating the unprecedented extent of crop introductions during this period.

**Supplementary Information:**

The online version contains supplementary material available at 10.1007/s00334-025-01060-9.

## Introduction

Historians and archaeologists have long been interested in the movements of crops (for a brief historiographic overview see Heinrich 2017, pp 144–149), which may catalyse or reflect broader changes in agricultural and food systems. The phenomenon by which a set or ‘package’ of new crops is introduced simultaneously, often alongside new technologies, is sometimes labelled an ‘agricultural revolution’. This has famously been argued by Watson (1974, 1981, 1983) to have happened in North Africa and the Levant following the Arab Conquest, though his Islamic Green Revolution (IGR), originally referred to as the “Arab Agricultural Revolution” and the “Medieval Green Revolution”, continues to be debated (cf. Decker 2009; Mir-Makhamad and Spengler 2023; Varisco and Fuks 2024). Meanwhile, the significance of the Roman Agricultural Diffusion (RAD) is becoming increasingly apparent (Fuks et al. 2023; cf. Heinrich 2017). Synthesizing the evidence for the earliest attestations of crop taxa, i.e. their ‘prevenience’ (Fuks et al. 2020a), should enable us to pinpoint the introduction and diffusion of each crop into the region, and allow us to better assess the RAD and IGR phenomena in the southern Levant.

As the diffusion of crops follows social, political, and cultural changes that do not neatly align with centennial or millennial demarcations, we define our period of study as ‘the long 1st mill. ce’, from the time of Alexander the Great in the late 4th century (c.) bce to the end of the Early Islamic period in the 12th c. ce. After a brief reflection on the study area, our sources, and some methodological considerations, we discuss the archaeobotanical and written evidence for new introductions to the crop curriculum of the southern Levant. In this, we start from the earliest occurrence of each taxon for the region from the Neolithic onwards, mentioning both the first archaeobotanical find as well as the first attestation in the written sources. This is essential as in order to establish which plants were introduced and/or became available for use during the long 1st mill. ce, we need to first ascertain which crops/plants were already present at the onset of the period. This requires a careful review and compilation of the available archaeobotanical and historical evidence, as no such overview currently exists. The resulting synthesis may serve as a starting point for this discussion, which future studies can build upon. We have organized the taxa we will discuss into six functional categories: (1) cereals; (2) pulses; (3) fruit and nut trees; (4) vegetables and tubers; (5) fibre, oil, and dye crops; (6) condiments, herbs, medicinal plants, and sweeteners.

## The southern Levant

The southern Levant includes the region comprised of modern-day Israel, Palestine, and Jordan. With the exception of a more temperate strip along the eastern Mediterranean, the region has a mostly semi-arid to arid climate, and this has generally been the case since the advent of the Holocene, notwithstanding climatic fluctuations between periods (e.g. Touchan et al. 1999; Rambeau 2010; Finné et al. 2011; Labuhn et al. 2016; Langgut and Finkelstein 2023). Some (micro-)regions in the southern Levant experience levels of precipitation that are on the limit of what is possible for arable agriculture (less than 200 mm per annum). Farmers of previous millennia developed a variety of strategies that would allow them to cultivate a wide range of crops even in these more arid areas, which has both been associated with the “greening of areas” through the expansion of agricultural zones in some areas and the (over-)exploitation of natural resources within others (e.g. Ramsay et al. 2016; Avni et al. 2019 and Langgut et al. 2021 for the Negev; see Heinrich and Hansen 2021 for a theoretical perspective). The zones of cultivation were expanded over time with the introduction and development of more advanced hydraulic systems, particularly in the “long” 1st mill. ce (e.g. Berenfield et al. 2016 and Ortloff 2020 for Petra; Rosen 2017; Avni et al. 2019; Fuks et al. 2020a for the Negev). These include the construction of several intricate types of water collection and distribution systems such as cisterns, canals, aqueducts, check-dammed plots, qanats, and plot-and-berm systems, variously introduced between the Roman and Early Islamic periods (see Lightfoot 1997, 2000; Abudanh and Twaissi 2010; Avni et al. 2019; Avni 2021; Roskin and Taxel 2021; Taxel and Roskin 2025). Overall, the 1st mill. ce bore witness to a particularly active period of agricultural expansion, experimentation, agro-technological innovation and intensification, especially as new crops entered the crop curriculum.

The southern Levant has a rich and pioneering history of botany, archaeobotany, wild plant resource management, agriculture, and domestication research (Zohary 1966, 1972; Feinbrun-Dothan 1978, 1986; Danin 2004; Fuller et al. 2011; Weiss and Zohary 2011; Mascher et al. 2016; Langgut 2024). The region also holds an important place in food history as it has yielded the first attestation of a precursor to bread made with wild cereals (Arranz-Otaegui et al. 2018). Simultaneously with, yet independently of, Egypt, Anatolia, and Mesopotamia, the southern Levant was among the first places where food processing and preservation techniques such as dairy and cereal-based product fermentations and long shelf life fruit products like olive oil are evident (e.g. Galili et al. 1997; Gregg et al. 2009; Aouizerat et al. 2019). The region also has a relatively long tradition in archaeobotanical research and hence there is a great wealth of studies that the current review and synthesis can draw upon, even though these are not evenly distributed over periods and (micro-)regions.

Historically, the southern Levant was a part of multiple changing polities, often in the same province or administrative region within a larger polity (e.g. Arabia Petraea [2nd–7th c. ce], Syria Palaestina [2nd–4th c. ce] and Palaestina Salutaris [4th–7th c. ce] during the Roman and Byzantine Empires, and Bilad al-Sham from the 7th–late 19th c. ce during the Rashidun Caliphate and subsequent Umayyad, Abbasid, Fatimid, Mamluk, and Ottoman Empires). Therefore, the socioeconomic and political substrate was likely commensurable for our main period of interest while technologies, ideas, and crops could move relatively freely within the region. This did not stand in the way of agricultural diversity, as the historical sources from different periods often refer to distinct crop specialisations in sub-regions. For example, in the 10th c. ce, al-Muqaddasī listed the various products and crops of Bilad al-Sham, highlighting local specialisations throughout the region. He stated, almost in a manner of local pride for his homeland, “that gathered together in the province of Palestine are 36 products not to be found together in any other land” (Collins 2001, p 152).

A rich body of written sources with information on agriculture and food exists for the region. For the first half of the 1st mill. ce, these especially consist of rabbinic texts, such as the *Mishna* (redacted in the 2nd c. ce), *Tosefta*, and *Jerusalem Talmud* (redacted 3rd–5th c. ce), while the works of Classical authors such as Flavius Josephus (*Ηistoria Ioudaikou polemou pros Rōmaious* and its later translation *De Bello Judaica* as well as his *Antiquitates Iudaicae)*, Pliny the Elder (*Naturalis Historia*), and Strabo (*Geographica*) play a secondary, complementary role. In the latter part of the 1st millennium, Arabic texts, such as the *Aḥsan al-taqāsīm fī maʿrifat al-aqālīm* (*The Best Divisions in the Knowledge of the Regions*) by the 10th c. ce geographer al-Muqaddasī and the *Kitāb al-Filāḥa al-Nabaṭiyya* (*Book of Nabataean Agriculture*) and *Kitāb al-Sumūm (Book of Poisons)* by the 10th c. ce agronomist Ibn Waḥshiyya, both of local origin, provide microregional descriptions of crops and agricultural techniques and strategies for the southern Levant. Al-Muqaddasī wrote lyrically in the *Aḥsan* about the agricultural performance and potential of Bilad al-Sham (i.e. Greater Syria):

“The region of Syria is splendid…This land provides for the needs of this world and the next; for here the heart is cheered and worshippers extend their bodies in prayer. Then of course there is Damascus, the paradise of this earth, and Sughar which is like a miniature Basra; beautiful Ramla, with its white bread and Īliyā (Jerusalem) the splendid, without tribulation; Hims is famous for low prices and excellent air; the mountain of Busrā with its vineyards should not be forgotten; nor Tiberius, renowned for its crops and its villages”.

Translation Collins 2001, p 128.

Indeed, such encyclopaedic works, including agricultural manuals (*filāḥa)*, geographies, cookbooks, tax registers and other written sources formed the basis of Watson’s (1974, 1981, 1983) argument for the Islamic Green Revolution (IGR, following Decker 2009).

## Methodological considerations

Our assessment of crop introductions and diffusion for the 1st mill. ce synthesizes diverse data across different disciplines and multiple archaeobotanical publications. Such an effort comes with many methodological challenges, of which we will consider some of the most important here (cf. Heinrich 2017; Fuks et al. 2020a, b). First, it should be noted that the numbers and types of sites, samples taken, and well-dated contexts pertaining to the 1st mill. ce are not homogenously distributed over the region and period. Furthermore, for most contexts only relative dates, that sometimes may span several centuries, are available, obscuring the timing of diffusion. The use of cultural periods comes with methodological challenges: most importantly their length is uneven; they are not always equally distinguishable in the archaeological record, while their precise chronological demarcation has been subject to debate and change. At the same time, they represent a standard unit of archaeological reckoning that helps us understand diachronic change. This situation may improve over time as more sites are studied and absolutely dated, and more distinct patterns may emerge, but for now we must treat the data as is. In addition, some taxa, for reasons of preservation, taphonomy, and the specific plant part used, are likely underrepresented in the archaeobotanical record—this especially applies to leafy vegetables, fleshy fruits, roots, and rhizomes. Therefore, banana or plantain (الموز), for instance, though mentioned as a crop of the southern Levant by al-Muqaddasī (de Goeje 1877, p 181; Collins 2001, p 152) is very unlikely to be encountered in the archaeobotanical record and has thus far not been attested in this region. Conversely, some of the crops we are interested in are not mentioned in the written sources or there is debate on the terminology of their identification (e.g. see discussion below on the artichoke). It is through combining the written sources and the archaeobotanical evidence that this paper aims to address the crops that neither discipline could tackle alone. Inevitably, such an approach remains methodologically imperfect, especially with respect to the uneven chronological distribution of different types of evidence. Written sources are more common for later periods. This is for instance due to the spread of literacy and the increased importance of writing throughout society, the interplay between the convenience and preservation of the writing materials used, as well as the more recent periods belonging to a continued historical tradition. Archaeological and consequently archaeobotanical studies, however, are more common for earlier periods due to a disciplinary bias in archaeological interest in the region (e.g. concerning crop domestication, the onset of agriculture, and Biblical archaeology). With respect to the archaeobotanical evidence, it could moreover be argued that due to the longer duration of the earlier periods, there might be a greater chance of encountering rarer plant remains, or those less likely to survive for taphonomic reasons. It cannot be reliably assessed to which degree these opposite biases may or may not cancel each other out. As so often in history and archaeology, we have to make do with the evidence we have, not the evidence we would wish for. Over time, as scholarship advances, some of these imbalances will become increasingly resolved. This, however, does not impede our aim, which is to view trends in crop use from the bird’s eye perspective.

Another consideration we wish to touch upon is that some first attestations of a taxon may represent the encounter of an isolated one-off specimen. Such specimens, rather than being indicative of a genuine introduction, may represent a local wild plant, an imported good, a temporary or failed attempt at the introduction of a crop, or in the worst case, a misidentification, contamination, or misdated sample. Besides the earliest occurrence of a new innovation, its widespread integration and embeddedness in the agricultural system is crucial in assessing its systemic and societal effects (van der Veen 2010). We will therefore contextualize isolated finds and in such cases also reflect on later periods when the taxon in question became more common. Contrariwise, the introduction and rise to dominance of new crops need not signify the sudden or complete disappearance of previously used crops, which may continue to play minor roles in specific functions or as part of broader diversification and risk-spreading strategies. Shifts in crop selection are therefore rarely clean breaks or determinate historical events, but rather gradual processes. In addition, part of the complexity of crop introductions may currently be beyond our immediate view. In archaeobotany, we can typically identify taxa to the (sub-)species level, whereas historical actors would have also interacted with crops on the level of the cultivar or landrace. Historical sources therefore often mention new, regional, or local ‘types’ of cereals and fruits with particular traits or uses (as al-Muqaddasī does in his passage on the products of Palestine for figs, raisins, apples, grapes, and plums), and while the diffusion of specific cultivars may have had profound economic effects, we cannot generally identify them in the archaeobotanical record, despite recent breakthroughs in this direction (e.g. Ramos-Madrigal et al. 2019; Wallace et al. 2019; Cohen et al. 2023; Landa et al. 2024; Meiri and Bar-Oz 2024).

A phenomenon that may also be observed in the data is that some native wild plants are encountered in the archaeobotanical record, which may have been actively managed or even cultivated, e.g. *Lathyrus ochrus* (Cyprus vetch) and *Coriandrum sativum* (coriander) (Frumin et al. 2015; Weiss et al. 2019; respectively). In addition, evidence is amassing that barley is native to the southern Levant and was domesticated there (Mascher et al. 2016; cf. Fuller et al. 2011). Another example is that of native *Indigofera* sp. taxa that were exploited in the 1st mill. ce and subsequently became important for the indigo dye industry (see below). We will refer to these plants as ‘Inno-native’ Crops or Food Plants and tag them as such in our data.

**Fig. 1:**
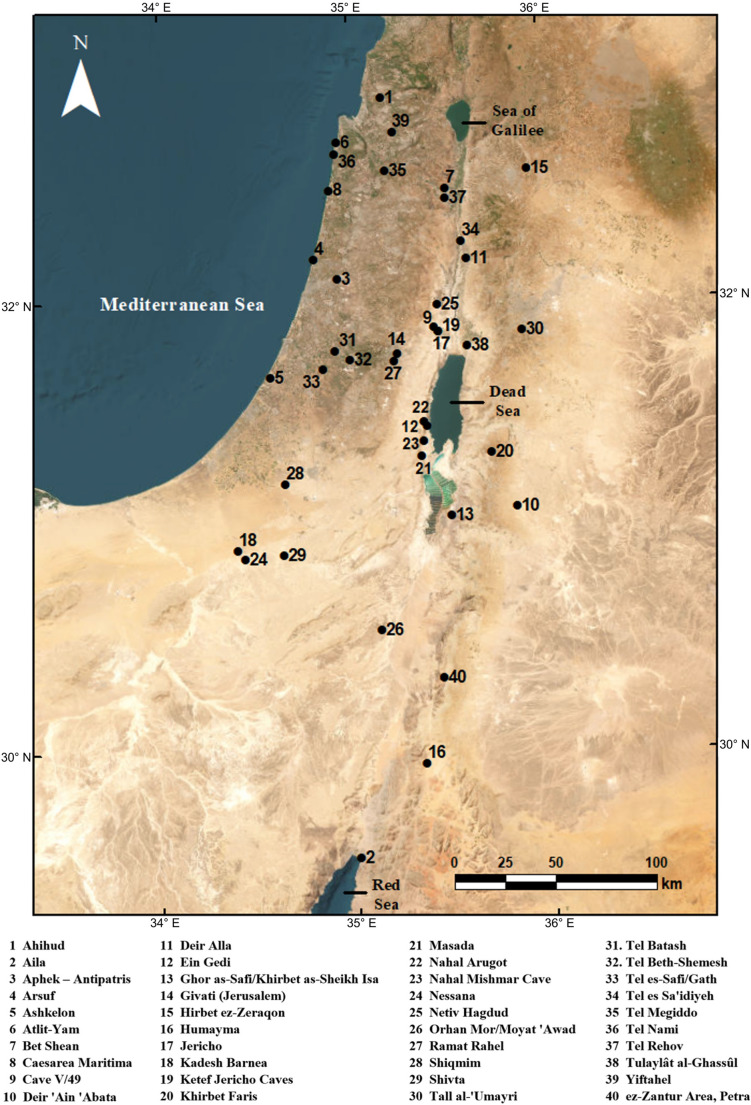
Map of archaeological sites with earliest attestations of crops in the southern Levant (Design by Gerardo Diaz)

To present a broad-brush analysis of 1st mill. ce contributions to agricultural diversity in the southern Levant, we compare the numbers of crops first attested in each of several key periods from the origins of agriculture up to the influx of crops from the Americas. To facilitate this effort, we tag crops as either (i) Neolithic Founder Crops, associated with the so-called “Neolithic Agricultural Revolution” (Zohary et al. 2012); (ii) Early Fruit Domesticates, associated with the plant component of the “Secondary Products Revolution” in the Chalcolithic period and reaching full entrenchment by the Early Bronze Age (EBA) (Weiss 2015; Fuller and Stevens 2019; Langgut 2024); (iii) the following Bronze–Iron Ages that saw some important additions to local crop repertoires, particularly in the 1st mill. bce (Frumin et al. 2015), (iv) RAD crops (1st c. bce–4th c. ce); and (v) IGR crops (latter 7th c. ce to 12th c. ce), with RAD and IGR representing two ends of the “long” 1st mill. ce (Fuks et al. 2023). Other periods in which the evidence for new introductions is minimal have been tagged as “Transitional/Other” for the purposes of this paper. Table 1 presents the periods covered by this synthesis, and the corresponding relative date ranges and crop ‘tags’. Note that tags follow cultural periods and established historical-agricultural phenomena, which differ in length. Some crops are assigned the tag “Indeterminate” as we do not have enough evidence from the written sources or archaeobotanical record to confidently assess the period of introduction at this stage. We present the full dataset of crops and information on their earliest attestations (historical or archaeobotanical) in the ESM. A map of the 40 sites in the southern Levant with the earliest attestations discussed in this paper is presented in Fig. 1.

**Table 1:** Chronological periods of plant domestication/introduction in the southern Levant

Period Tag	Relative Date Range (centuries)	Cultural Periods	Crop Tag
Late Medieval	12th–16th ce	Seljuk, Crusader, Ayyubid, Mamluk, Ottoman	Transitional/Other
Early Islamic	7th–12th ce	Umayyad, Abbasid, Fatimid	IGR
Byzantine	4th–7th ce	Byzantine	Transitional/Other
Roman	1st bce–4th ce	End of Hellenistic, Roman	RAD
Persian-Hellenistic	6th–1st bce	Babylonian, Persian, Hellenistic	Transitional/Other
Iron Age II	11th–6th bce	IA II A, B, C	Bronze-Iron Age Introduction
Late Bronze-Iron Age I	16th–11th bce	LB I, IIA, IIB; IA IA, IB	Bronze-Iron Age Introduction
Middle Bronze Age	20th–16th bce	MB I, II, III	Bronze-Iron Age Introduction
Early Bronze Age	36th–21st bce	EB I-IV	Early Fruit Domesticate
Chalcolithic	45th–36th bce	Early/Late Chalcolithic (Ghassulian)	Early Fruit Domesticate
Pottery Neolithic	65th–45th bce	PN A, B	Neolithic Founder Crop
Pre-Pottery Neolithic B	88th–65th bce	PPNB	Neolithic Founder Crop
N/A	N/A	N/A	Indeterminate
N/A	N/A	N/A	Inno-native Crop/Food Plant

### Cereals

The most important cereal crops from the Neolithic onward are wheats and barleys. During the Neolithic, *Triticum turgidum* ssp. *diccocum* (emmer wheat) was domesticated and its earliest attestation in the southern Levant is from Jericho (Hopf 1983). It remained an important cereal crop in the region well through the Iron Age [e.g. LBA Tell es-Safi and Tel Miqne (Frumin et al. 2019) and Iron Age (IA) Deir Alla (van Zeist and Heeres 1973 and Neef 1989)]. *Triticum monococcum* ssp. *monococcum* (Einkorn wheat) is attested slightly later at Neolithic Jericho (middle PPNB), but it never became an important crop in the southern Levant (Hopf 1983; cf. discussion in Zohary et al. 2012, p 38)^1^. The earliest attestation of tetraploid free-threshing wheat (*Triticum turgidum* s.l.), as evidenced through its rachis, is from Chalcolithic Shiqmim (5000–3200 bce) (Kislev 1987). It has, however, been hypothesized based on seed morphology that tetraploid free-threshing wheats may have already spread across southwest Asia during the Neolithic (Kislev 1979; Kislev 2009). *Triticum aestivum* ssp. *aestivum* (free-threshing hexaploid wheat), as evidenced through its rachis, has been attested from the Late Byzantine period onwards at Shivta/Isbeita and Nessana/Auja al-Hafir (Fuks et al. 2023). 

Barley, both the two-rowed (*Hordeum vulgare* ssp. *vulgare* var. *distichum*) and six-rowed (var. *hexastichum*) varieties (particularly the hulled types, though naked/free-threshing types were likely also present), remained very important in the crop curriculum of the southern Levant throughout its history. It has been proposed that barley may have been locally domesticated in the Upper Jordan Valley, and therefore it may be considered an Inno-native Crop/Food Plant, while simultaneously representing an important Neolithic founder crop (Mascher et al. 2016; cf. Fuller et al. 2011). The earliest evidence from the southern Levant for two-row (hulled) barley comes from Neolithic Jericho and that for six-row barley from Chalcolithic Shiqmim (5000–3200 bce) where it was found in small quantities (Hopf 1983; Kislev 1987). Six-row barley is also attested at EBA Jericho and is potentially attested at EBA Ḫirbet ez-Zeraqōn (2500–2250 bce) in the southern Levant and EBA and MBA Tell Mozan (Urkesh) in the northern Levant (Hopf 1983; Riehl 2004, 2010), with two-row barley appearing to be dominant at these sites and periods. It therefore seems that both varieties of barley became part of the crop curriculum very early in both the southern and northern Levant (cf. Zohary et al. 2012, pp 51–58). In historical debates, barley is often underestimated as a crop and often relegated as a foodstuff for the poor, religious ascetics, an ingredient for beer making or animal fodder. Barley, however, was also an important ingredient in bread-making and for a variety of other foodstuffs, while it is also mentioned in the written sources as a unit of measure for the weight of currency, for comparing units of weight between regions, and for calculating taxes (al-Muqaddasī (de Goeje 1877, p 182; Collins 2001, p 152). Millets are not frequently found in the archaeobotanical record of the southern Levant. Broomcorn millet)* (Panicum Panicum)* has been potentially attested from the EBA at Ḫirbet ez-Zeraqōn (Riehl 2004 who identified it as *Panicum *cf. *miliaceum*) and *Setaria italica* (foxtail millet) was attested later in the Middle Nabataean to Byzantine period (mid-1st–4th c. ce) at Humayma (Ramsay 2013). *P. miliaceum* has been attested later in the MBA at Mozan (Urkesh) in the northern Levant (Riehl 2000 identified as cf. *P.* *miliaceum*) and in the Early Iron Age at Khitbat al-Mudayna al-‘Aliya (Farahani et al. 2016). *Panicum* sp. (though the authors state ‘broomcorn millet’ in the text) has also been attested later in Late Bronze Age/Iron Age I (LBA/IA I, 1300–1200 bce) and IA II//Persian (550–400 bce) at Tall al-‘Umayri (Ramsay and Mueller 2016) and much later in Mamluk Tall Abu Sarbut and Ottoman al-Humayma, though also in small numbers (Grootveld 2008 and Ramsay 2013; respectively). In the text, Ramsay 2013 refers to the caryopses from her study at Humayma as *Setaria* sp. (though specifies them as ‘foxtail millet’). Ramsay and Smith 2013 have also found millets in Byzantine contexts at Bir Madhkur, though these specimens were designated as *Setaria* sp./*Panicum* sp. *Setaria italica* has been attested much later in an early Ottoman period (16th c. ce) context at Khirbet Beit Mazmil (Hansen in press). This evidence suggests that farmers from the Bronze Age on were already familiar with cultivating both summer and winter crops, though Watson suggests that summer crop cultivation only became more common during the Early Islamic period (Watson 1983, pp 103–107). Another summer crop, *Sorghum bicolor* (sorghum), is thought to have been already known and cultivated in other parts of the Islamic world by the 10th c. ce (Watson 1983, pp 12–14); however, it seems to have been introduced into the southern Levant slightly later. It is for instance attested by an 11th c. ce exegete of the *Mishna*, and in other written sources from the 13th c. ce (Amar 2000, p 78). Archaeobotanical finds of sorghum have been encountered in contexts dating to the 13th c. ce at Khirbet Faris and in the 14th c. ce at Tall Abu Sarbut (Hoppé 1999; Grootveld 2008; Hansen 2012), also suggesting an introduction sometime after the 10th c. ce. A potential sorghum caryopsis was also found in a Mamluk context at Dhiban (Farahani 2018). By the 18th c. ce, the traveller Carsten Niebuhr wrote that sorghum had become a “staple of the peasants” in both Syria and Palestine (Watson 1983, p 14). Scholars have variously interpreted the biblical *dohan* (דחן), mentioned as part of the multi-grained bread eaten by Ezekiel (4:9), as either broomcorn millet, foxtail millet, or sorghum (Feliks 1968, p 154). On philological grounds, as well as the archaeobotanical evidence, broomcorn millet is the most likely translation, although it is possible that *dohan* was a general term for millets (Amar 2012, p 130). In the *Mishna* (*Shevi’it* 2:7), Feliks (1980, pp 128–129) identifies *dohan* (דוחן) as broomcorn millet (acknowledging foxtail millet as a second-choice candidate) and *pragin* (פרגין) as sorghum, which are mentioned together with sesame and rice—all of which were summer crops in the region. *Oryza sativa* (rice) has not been attested yet in the archaeobotanical record in the southern Levant; it was however well attested in local written sources, from the *Mishna* onwards (see Spengler et al. 2021 for a review).

### Pulses

Pulses, like cereals, were important staples in the southern Levant throughout history. Use of their wild progenitors as a food source has been attested at Palaeolithic sites including one in Jordan possibly representing early archetypes of ‘bread’ or ‘porridge’ (Arranz-Otaegui et al. 2018; cf. Kabukcu et al. 2023). In the southern Levant, various pulses were cultivated from early on in the Neolithic; these included *Cicer arietinum* (chickpea), *Lens culinaris* (lentil), *Pisum sativum* (pea), and *Vicia faba* (broad bean) respectively at Jericho, Yiftahel, and Ahihud (Hopf 1983; Kislev et al. 2012; Caracuta et al. 2017). A recent study on the wild progenitor of *V. faba* suggests its early use in the southern Levant at the Natufian site of el-Wad Terrace long prior to its domestication (Caracuta et al. 2016). At Ahihud, the authors interpreted the numerous specimens of broad bean as domesticated, while they were uncertain as to the domestication status of their *Lens *and *Pisum *specimens; they did conclude, however, that the variety of pulses found at Ahihud indicates “an earlier stage of legume domestication, when farmers were experimenting with different species, some of which would eventually become staple crops” (Caracuta et al. 2017, p 15). From the aggregate archaeobotanical evidence, it is clear that additional Fabaceae were introduced or added as Inno-native Crops/Food Plants to the crop curriculum during the MBA and LBA. These include *Vicia ervilia* (bitter vetch) (Kislev 1993; Simchoni et al. 2007; Weiss et al. 2019), *Lathyrus clymenum* (Spanish vetchling) (Kislev et al. 1993), *Lathyrus sativus/cicera* (grass pea/red vetchling)^2^ (Kislev et al. 2006a; Ramsay and Mueller 2016), and *Trigonella foenum-graecum* (fenugreek) (Weiss et al. 2019). While fenugreek is often interpreted as a fodder crop, condiment, or medicinal plant, at least during the 1st c. ce it appears to have been a pulse used for porridges, as famously mentioned at the siege of Jotopata in 67 ce (Josephus *De Bello Judaica* 3.7.29). In addition, the local pulse *Lathyrus ochrus* (Cyprus vetch) was used as a food plant during the LBA, though its use has not been documented for other periods (Weiss et al. 2019). We consider the Cyprus vetch an Inno-native Crop/Food Plant, as the taxon is native to the southern Levant and was deliberately exploited as a food plant during this period, though its exploitation later seems to have discontinued. This pattern is also seen with other Inno-native Crops/Food Plants, e.g., the exploitation of *Lupinus pilosus* (blue lupine) for food during the Epipalaeolithic (Natufian) at the Hayonim Cave in the western Galilee (Hopf and Bar-Yosef 1987), and that of *Vicia peregrina* (rambling vetch) at Neolithic Netiv Hagdud and Bronze Age Tel Rehov, which has been interpreted as a food plant (Melamed et al. 2008; Caspi et al. 2020).

During the 1st mill. ce, additional pulses start to appear both in the written sources and archaeobotanical record. One of these is *Vigna unguiculata* ssp. *unguiculata* (cowpea or black-eyed pea), which was likely introduced in the wider Mediterranean sometime between the Late Roman and Early Islamic periods (Heinrich and Wilkins 2014). Its first archaeobotanical find in the southern Levant dates to the mid-10th–12th c. ce at Ashkelon (Forste et al. 2022). Archaeobotanical finds from the northern Levant in the form of cotyledons of cowpea have been attested slightly earlier, from Abbasid (8th–9th c. ce) Tell Guftan followed by Seljuk/Zengid Qaryat Medad (early 12th–third quarter 12th c. ce) in the Euphrates Valley (Samuel 2001). The cowpea was depicted somewhat earlier in the Byzantine herbal *Juliana Anicia Codex* (512 ce; illustration 370v, see Janick and Hummer 2012; Fig. 13), which is a richly illustrated later copy of the (now lost) 1st c. ce pharmacopeia *De Materia Medica* by Pedianus Dioscorides. Contemporary to the *Juliana Anicia Codex* is the travelogue by the anonymous Piacenza Pilgrim (570 ce), who described a two-foot long “beanstalk” (*‘virga fasiola’*), likely referring to the length of the pod or fruit, that he saw in Jericho (cf. Stewart 1887, p 13; Amar 2000). Aubrey Stewart, in his translation of the travelogue from 1887, suggested *Ceratonia siliqua* (carob) as its identification, but as its pods only measure between 10 and 30 cm (Batlle and Tous 1997; Shepperd 2008), the description seems to fit *V. unguiculata* ssp. *sesquipedalis* better; this is a subspecies of cowpea, referred to as the yard-long bean, and it is eaten as a green vegetable.

Another 1st mill. ce introduction was *Lupinus albus* (white lupine). The first written source evidence originates from the *Mishna* (cf. Löw 1967), while the first archaeobotanical evidence was encountered at 7th–8th c. ce Nessana (Fuks et al. 2023). In the 10th c. ce, al-Muqaddasī mentions lupine as one of the products of Palestine (Collins 2001, p 152). The written sources suggest that *Vigna radiata* (mung bean) and *V. mungo* (black gram or urid bean), both of which originate from the Indian subcontinent, may also have been Early Islamic introductions into the southern Levant. Amar and Lev state that both were associated with the Arabic word *mash* and that the earliest mention in the Arabic sources is by 10th c. ce Arab Andalusian pharmacologist Ibn Juljul (Amar and Lev 2018, p 68), while the earliest mention explicitly for the southern Levant is in an 11th/12th c. ce commentary on the *Mishna* (Amar 2000). In the 13th c. ce, Ibn al-Baytar, an Arab Andalusian physician and botanist who travelled widely and collected specimens from (southern) Bilad al-Sham, mentions *mash* soup as a remedy for coughs and colds in his *Compendium on Simple Medicaments and Foods* (Nasrallah 2010, p 799).

### Fruit and nut trees

Arboriculture and the exploitation of wild tree resources have a long tradition in the southern Levant. Though some of the taxa in the latter group were exploited from very early on, it remains challenging to pinpoint the moment in time from which they were actively managed, cultivated, or domesticated—for instance in the case of *Ficus carica* (fig) (see Kislev et al. 2006b; Weiss 2015; Langgut and Garfinkel 2022; Whitlam et al. 2023). Wild tree taxa such as *Pistacia atlantica* (Atlas pistachio, Kislev 1988a, 1997; White and Makarewicz 2012; Asouti et al. 2015) and local oak species (e.g. *Quercus* cf. *ithaburensis*, Kislev 1997) were also exploited from early on. Among the earliest tree crops to be cultivated are *Olea europaea* (olive), *Vitis vinifera* (grape vine), *Phoenix dactylifera* (date palm), *Ficus carica*, and *Punica granatum* (pomegranate) (for comprehensive reviews, see Abbo et al. 2015; Weiss 2015; Langgut 2024; cf. Besseiche et al. 2025). Until recently, it was assumed that pomegranate is not native to the southern Levant and was introduced to the region only during the EBA. Yet, a new review study shows that pomegranate remains have been present at archaeological sites in the region since the Early Palaeolithic, though in relatively low frequencies (Langgut 2024). Once incorporated into the crop curriculum during the Chalcolithic and EBA, these species would remain core crops in the southern Levant up to this day. *Prunus amygdalus* (almond) is considered to be part of this group, although the available evidence does not facilitate pinpointing the timing of its active cultivation and domestication (Zohary et al. 2012, pp 147–148).

The earliest attestation of the fruit of the *Ficus sycomorus* (sycamore fig) originates from EBA (4,900 BP/2950 bce) Tell es-Sa’idiyeh (Cartwright and Clapham 1993; Cartwright 1997), and its wood was attested once in a LBA I context at Tel Beth-Shean (Baruch 2007). The sycamore fig seems to become more common during the IA, and evidence suggests it was exploited for both its fruit at Ashkelon (12th c. bce) as well as its wood resources at Tel Rehov (10th–9th c. bce) (Frumin et al. 2015; Liphschitz 2020). Wood from the sycamore fig may have been imported into the region during the BA or earlier for use as timber (see discussion in Frumin et al. 2015). The sycamore fig remained an important crop in the region and was mentioned by al-Muqaddasī as the “sycamore fruit” (االجمّيز) in the 10th c. ce (Collins 2001, p 152). *Juglans regia* (Persian walnut) was introduced into the southern Levant during the MBA, while *Morus nigra* (black mulberry) might have been exploited for wood resources from the IA onwards (Liphschitz 2000, 2020; Langgut 2015). Evidence for cultivation of the black mulberry next appears in written sources from the Hellenistic period (*Book of the Maccabees*) and the *Mishna* as *tut* (תות – 1st c. bce—2nd c. ce). In archaeobotany, black mulberry nutlets appear in Early Islamic contexts at the Givati parking lot site in Jerusalem (Amichay and Weiss 2020). 

During the Persian period, *Citrus medica* (citron) is convincingly attested in the local crop curriculum (Langgut 2015). *Pinus pinea* (stone pine) and *Ceratonia siliqua* became cultivated crops by the late Hellenistic period (1st c. bce; Kislev 1988b; Zohary 2002). In the 10th c. ce, al-Muqaddasī praises the quality of pine nuts from the region, which he says is referred to locally as the “*Quraysh-Bite*” (Collins 2001, pp 151–152). While it is a component of the local wild vegetation, and frequently attested at archaeological sites, the carob seems to have been domesticated only in the Hellenistic period (Zohary 2002). The earliest chronologically secure attestation of *Malus domestica* (apple) from the archaeobotanical record comes from IA Kadesh Barnea/Qadesh Barne‘a/Tell el-Qudeirat (Kislev unpublished in Zohary et al. 2012, p 137 and Amichay and Weiss 2020, p 659) and is followed by mid-8th–9th c. ce finds of apple from Jerusalem (Amichay et al. 2019). Written source evidence for the apple, however, has a much longer tradition going back to biblical and rabbinic texts, demonstrating that while the apple might not have been cultivated in the southern Levant until the Roman period, it was apparently known in the region before then (Feliks 1968, p 60; Amar 2012, pp 122–123; Amichay and Weiss 2020 for a review). 

During the Nabatean and Roman periods, many new fruit trees enter into the southern Levant crop curriculum. *Corylus avellana* (hazelnut), *Cydonia oblonga* (quince), *Pistacia vera* (pistachio nut), *Prunus domestica* (plum), *P. persica* (peach), *P. armeniaca* (apricot), *Pyrus communis* (pear), and *Ziziphus jujuba* (Chinese date/true jujube) are all introduced during this period. There is archaeobotanical evidence of *Corylus* sp. from both the Roman period at Nahal Arugot and from the Byzantine–Early Islamic period at Shivta (Kislev and Simchoni 2006; Langgut et al. 2021). Pollen of *Corylus* sp. dated to the Roman period was also found in Caesarea Maritima, though suggested to be used for ornamental purposes (Langgut et al. 2015). In the same period, hazelnut is associated with the term *ilsarin* (אילסרין) in the *Tosefta* and *pundekin* (פונדקין) in the *Jerusalem Talmud* (Löw 1967; Feliks 1994, pp 248–250). The quince is confidently identified in rabbinic texts from the Roman period onwards (Feliks 1994, pp 219–221). The earliest archaeobotanical attestations of *Pistacia vera* come from the Roman period in the caves of Ketef Jericho and later in the mid-7th–8th c. ce at Shivta (Hartman and Kislev 1998; Fuks et al. 2023). These are complemented by rabbinic references which appear to refer to *P. vera* rather than cultivated local wild pistachio (Feliks 1994, pp 174–176). For the plum, both written sources (the *Mishna*) and the archaeobotanical evidence (from Masada) place the diffusion and integration of the taxon into the crop curriculum during the Roman period (Tabak 2006). The peach appears in written sources from the Roman period (Feliks 1994, pp 229–232), which is contemporary with the earliest finds of archaeobotanical peach pits from Orhan Mor/Moyat ‘Awad (Kislev and Simchoni 2009). Peach is also attested archaeobotanically in the 1st mill. ce at Petra (1st half of the 4th c. ce), at Deir ‘Ain ‘Abata (5th–6th c. ce), at Caesarea Maritima (4th–6th c. ce), at Elusa (4th-mid–5th c. ce), and in Late Byzantine–Early Islamic Shivta and Nessana (mid-6th–8th c. ce) (Ramsay 2010; Hoppé 2012; Bouchaud et al. 2017; Fuks et al. 2023; respectively). The apricot, which is recorded in written sources dating to the Early and Middle Islamic periods (Amar 2000), has been documented archaeobotanically during the 1st half of the 4th c. ce in Petra (Bouchaud et al. 2017) and in Byzantine Caesarea Maritima (4th–6th c. ce, Ramsay 2010). An endocarp identified as *Armeniaca* sp. is listed in a table of miscellaneous finds from an IA context from the City of David, but it may have been intrusive and was not included in the list of seeds and fruits in the authors’ report (Liphschitz and Waisel 1992, pp 106, 120). While terms associated with both the native wild Syrian pear (*Pyrus syriaca*; *khizrar* in Hebrew) and the common pear (*Pyrus communis; agas*, *crustomolin* in Hebrew) are mentioned in the *Mishna* during the Roman period (Feliks 1982), there has only been archaeobotanical evidence for the Syrian pear. This evidence spans the Epipalaeolithic to the Early Islamic periods (Amichay et al. 2019; Amichay and Weiss 2020 and references therein). The earliest archaeobotanical find of Chinese date/jujube originated from Early Islamic Shivta (Fuks et al. 2023), although there is a clear historical reference to it from the *Mishna* from the 2nd c. ce (Feliks 1994, pp 251–253). Later, in al-Muqaddasī’s 10th c. ce work, in a passage on the (food) products of Palestine, the term ‘*anaab* (العنّاب) is translated as jujube by Collins (2001, p 152). Today *‘anaab* is the Arabic term for the Chinese date, and the term is used by medieval exegetes as a translation for the Hebrew term *shizafin* (שיזפין) from the *Mishna* and other rabbinic texts (Amar 2000). The earliest attestation of *Mespilus germanica* (medlar) comes from the archaeobotanical record from Byzantine contexts at Caesarea Maritima dating to the 4th–6th c. ce (Ramsay 2010). Löw (1967) believed it was associated with the term *farish* (פריש) of the *Mishna* and the *merpaita*/*mespaita* of the *Jerusalem Talmud*. However, Feliks (1994, pp 219–220, 264) rejects the identification of these terms as medlar, and the unprecedented archaeobotanical remains from Caesarea Maritima probably represent exotic imports.

During the Early Islamic period, another set of fruit trees were evidently brought into the region for cultivation, including *Castanea sativa* (chestnut), *Citrus aurantium* (bitter orange), *C.* x *limon/aurantifolia* (lemon/lime), *Morus alba* (white mulberry), *Musa* sp. (banana/plantain), and *Prunus avium/cerasus* (cherry); we have designated these as IGR crops, since their introduction coincides with the Islamic Green Revolution as defined by Watson (1983). It should be noted that chestnut, white mulberry, and cherry were not a part of Watson’s original thesis, but the latter two are listed by Amar (2000, p 334) among the Early Islamic introductions to the southern Levant. Chestnut and ‘winter’ cherry were mentioned by Ibn Wahshiyya in the 10th c. ce, though it is uncertain if he is referring to *Prunus cerasus* (sour cherry) or *P. avium* (sweet cherry), so we have indicated both in the table (ESM; Levey 1966; Amar 2000, pp 222, 243–244). Ibn Wahshiyya, al-Biruni, and Ibn al-Baytar refer to the *mahlab* (*Prunus mahaleb*) or perfumed cherry in the 10th, 11th, and 13th centuries ce respectively, and note that it was used for the production of an oil for cooking and for medicinal, washing, and cosmetic purposes (Amar and Lev 2018, pp 114–115). In the early 12th c.ce, there is also a mention of cherry at Hebron by Daniel the Russian during his pilgrimage between 1106 and 1108 ce (Amar 2000, pp 243–244). The introduction of white mulberry almost certainly post-dates that of black mulberry (*Morus nigra)*, and it is assumed to accompany the introduction of silk production between the 6th and 9th centuries ce (Amar 2000; Amichay and Weiss 2020). Textual evidence suggests it became more common around the 11th c. ce (Fuks et al. 2020a), and the earliest archaeobotanical evidence comes from a tentative identification (*Morus* cf. *alba*) from Crusader period Arsur (Orendi et al. 2023). Banana/plantain is explicitly mentioned by al-Muqaddasī as one of the crops from Jerusalem and the Ghawr (Collins 2001, pp 141, 147), but it has not yet been attested in the archaeobotanical record. Citrus fruits, with the exception of the citron, which was introduced earlier, have not yet been identified in the archaeobotanical record in the southern Levant. They have however been identified in the written sources of al-Muqaddasī (bitter orange) and Ibn Wahshiyya (bitter orange) in the 10th c.ce, Nassar Khusraw (lemon/lime) in the 11th c. ce, and an anonymous crusader (pomelo, *Citrus maxima*) in the 13th c. ce (Watson 1983; Grabois 1984; Amar 2000). For some fruit trees, such as the *Cocos nucifera* (coconut), *Mangifera indica* (mango), and *Prunus avium* (sweet cherry), it is unclear how widespread they had become by the end of the 1st mill. ce. These fruit trees are moreover quite particular in terms of their environmental requirements. We may glean from both the written and archaeobotanical sources, however, that coconut was known and traded in the Middle East and Mediterranean already in Roman times (Amar 2000; Cappers 2006). Coconut is mentioned in the 10th c. ce as a medicinal plant by Ibn Wahshiyya in his *Book of Poisons*, which implies that it was at least accessible in markets in the southern Levant (Levey 1966).

### Vegetables and tubers

Vegetables and tubers were important field/garden crops; they are often mentioned in written sources, and a variety of them have appeared in the archaeobotanical record. However, some taxa have been more challenging to identify, as the plant parts and tissues that were used by humans (e.g. leaves, fruits, bulbs, rhizomes, or roots) typically preserve poorly, especially if they were processed or passed through the human digestive tract, while their seeds often do not enter or are not deposited in cooking or waste contexts. Mineralized preservation in previously waterlogged contexts, such as wells and cesspits, while rare for the region, provide evidence for the consumption of those plant parts, such as at Givati (Amichay et al. 2019; Amichay and Weiss 2020). It should be noted that also in cases where seeds are available, especially in low quantities, it is often difficult to determine whether they represent cultivated or wild specimens (e.g. onion and garlic from Chalcolithic Naḥal Mishmar; Zaitschek 1961, 1980), and whether they represent deliberate exploitation or only their presence in the local environment. 

In southern Levantine archaeobotany, *Daucus carota* (carrot) was first encountered at Neolithic Atlit-Yam (Kislev et al. 2004; Hartmann-Shenkman et al. 2015), but its usage and domestication status at this stage is unknown. Several millennia later, it is depicted in Byzantine herbals such as the *Juliana Anicia Codex* and the *Codex Neopolitanus* (Janick and Hummer 2012; Fig. 12; Janick and Stolarczyk 2012; Fig. 5). From the EBA onwards, remains of *Allium ampeloprasum* var. *porrum/A. ascalonicum* (leek/shallot) were found in Jericho, though these could not be brought down to a lower taxonomic level (Hopf 1983). One seed of *Brassica nigra* (black mustard) was found at PPNA Netiv Hagdud (Kislev 1997), while another potential specimen was found at EBA Jericho (Hopf 1983). Mustard was a cultivated crop by the Roman period, as documented through multiple references in the *Mishna* and *Jerusalem Talmud* (Stiebel 2018). *Lepidium sativum* (garden cress) was attested archaeobotanically at IA Deir Alla (Neef 1989) and Early Islamic Givati, while it is also mentioned in the *Mishna* (Amichay and Weiss 2020). *Cichorium intybus* (chicory) and *C. endivia* (endive) are native to the southern Levant. The earliest archaeobotanical find of endive in the southern Levant originates from LBA Tell es-Safi/Gath (late 16th–early 12th c. ce) (Frumin et al. 2019). Chicory has not been attested in the southern Levant, and the only find from the wider region thus far comes from MBA Tel Mozan (Urkesh) in the northern Levant (Riehl 2010; identified as *Cichorium* cf. *intybus*). Chicory and endive are mentioned in the 10th c. ce *Geoponika* in Book 12, associated with the classical Greek word *seris* and the medieval Greek word *troxima*, citing Didymos (1st c. bce–1st c. ce; Dalby 2011, pp 246, 262). They are also mentioned alongside several other (green) vegetables in the *Geoponika*, including leek, carrot, coriander, *Brassica oleracea* (cabbage), *B. rapa* (turnip), *Asparagus officinalis* (asparagus), *Raphanus raphanistrum* ssp. *sativus* (radish), *Cucumis melo* (musk melon), *Mentha* sp. (mint), *Allium cepa* (onion), *A. sativum* (garlic), *Cynara cardunculus* (artichoke) (see discussion below), and *Lactuca sativa* (lettuce) (Dalby 2011, pp 246–267). In the 10th c. ce, Ibn Wahshiyya in his *Book of Poisons* mentions that the leaves and the seeds of lettuce, chicory, endive, and the wild chicory are ingredients for antidotes (Levey 1966).

In the Roman period, additional vegetables enter both the archaeobotanical record and the written sources. Vegetables attested in the *Mishna* (1st c. bce–2nd c. ce) include the musk melon, radish, *Lagenaria siceraria* (bottle gourd), cabbage and turnip (Löw 1967; Feliks 1982; Amar 2000). Of these, only bottle gourd, musk melon, and radish have been attested archaeobotanically for the southern Levant, the former dating to the 1st c. ce at Moyat Awad/Orhan Mor and the latter two to the Early Islamic period at Givati (Kislev and Simchoni 2009; mid-8th–10th c. ce Amichay and Weiss 2020). The musk melon is also mentioned in the *Geoponika* for its medicinal applications, citing Florentinus (4th c. ce, see Dalby 2011, p 258). Similarly, radish is mentioned in various written sources, including the *Mishna* in the 2nd c. ce where it is listed as both a medicinal and edible plant, as well as in the *Geoponika*, again citing Florentinus (Dalby 2011, pp 259–260). In addition, radish is depicted in the Late Antique *Juliana Anicia Codex* (Janick et al. 2013; Fig. 3), and the same is the case for *B. rapa* (Janick et al. 2013; Fig. 3). The introduction of bottle gourd into the southern Levant likely predates the 2nd c. ce, as it was depicted on Egyptian wall paintings from the 18th Dynasty, 1550–1300 bce (Janick et al. 2007; cf. Keimer 1924). Archaeobotanical evidence for bottle gourd in the southern Levant comes from Nabataean–Roman Orhan Mor/Moyat ‘Awad (Kislev and Simchoni 2009). Domesticated *Citrullus lanatus/vulgaris* (watermelon) was cultivated in nearby Egypt during the BA, and the identification of *avatihim* in the Bible (Numbers 11:5) as watermelon is secure (Amar and Lev 2011). Hence, watermelon was probably known in the southern Levant in the BA or IA, although sweet varieties are first attested in the *Mishna* (Paris 2015, 2023). One watermelon seed was identified in a Byzantine context from Deir ‘Ain ‘Abata (Hoppé 2012), and watermelon features in various Byzantine mosaics (Avital and Paris 2014). In the Early Islamic period, the textual record suggests a proliferation of cultivars (Amar 2000; Paris 2015). In the archaeobotanical record, it is encountered in greater numbers in 13th c. ce contexts at Khirbet Faris (Hoppé 1999; Hansen 2012).

During the Early Islamic period, additional vegetables appear to enter the crop curriculum. Crops from this category attested in written sources include *Asparagus officinalis*, *Cucumis sativus* (cucumber), *Colocasia esculenta* (taro), and *Solanum melongena* (aubergine). While each of these crops is mentioned for the southern Levant in 10th c. ce works of both al-Muqaddasī and Ibn Wayshiyya (Levey 1966; Collins 2001; Amar and Lev 2011; Paris et al. 2012), only the aubergine (*bādhinjān*) has been attested archaeobotanically thus far, specifically at mid-8th–10th c. ce Givati and at 8th–9th c. ce Shivta (Amichay et al. 2019; Fuks et al. 2023; respectively). Asparagus might have been exploited earlier, as it occurs in the wild in much of the Mediterranean and is known to have been popular among the Romans. The Roman agronomists Cato the Elder (2nd c. bce), Columella (1st c. ce), and Palladius (4th c. ce) recommended collecting this plant from wild or semi-wild managed populations, while Columella even suggests transplanting specimens from the wild into the vegetable garden (see Frayn 1979 for a discussion). Asparagus is also mentioned in the 10th c. ce version of *Geoponika* (Dalby 2011, p 256). Like asparagus, taro may also have been present prior to the Early Islamic period. Taro (*kolkas*) is mentioned in the *Mishna* and *Jerusalem Talmud* in contexts suggesting it was a common local crop (Feliks 2005, pp 260–261; Grimaldi et al. 2018). *Colocasia *is furthermore mentioned in various Greek and Roman sources, though its identification as taro is not always clear (Grimaldi et al. 2018). Later there is an illustration of it in the herbal Morgan 652 that was produced in Constantinople between 927 and 985 ce (Janick et al. 2013; Fig. 7), but the manuscript is held to be a compilation of older herbals such as the aforementioned 6th c. ce *Juliana Anicia Codex* and the 7th c. ce *Codex Neopolitanus* (Janick et al. 2013). It constitutes the earliest known depiction of taro as a crop that we are aware of; the study of these codices and their compilation and editing history might further illuminate the diffusion of taro in the wider Mediterranean.

Two additional vegetables that might also have been introduced into the southern Levant during the Early Islamic period are *Cynara cardunculus* (cardoon) and *C. cardunculus var. scolymus* (artichoke). Neither of these has been attested archaeobotanically thus far, however, and the terminology for their identification in the written sources is still debated (Wright 2009). Collins has translated the term ‘*akkoub* (العكّوب) from al-Muqaddasī’s list of the products of Palestine as the artichoke (Collins 2001, pp 151–152). The term ‘*akkoub*, however, might refer to another native wild thistle, *Gundelia tournefortii* (tumble thistle), which is used as both a food and medicinal plant (Lev-Yadun and Abbo 1999; Halabi et al. 2005; Asadi-Samani et al. 2013; Barreiro 2019). In other Arabic written sources from the Early and Middle Islamic periods, for instance in the works of Ibn Wahshiyya, Ibn al-Baytar, Abu Hanifa al-Dinawari, Abu al-Khayr, and Ibn al-‘Awwam, the terms *kharshuf*, *qinīriyya*, and *kankar* have been associated with the artichoke instead of *‘akkoub*, while these three terms are not present in al-Muqaddasī’s work (de Goeje 1877). Meanwhile *kankar* (كنكر) has been argued by both Watson (1983) and Wright (2009) to be associated with the cardoon and not the artichoke, though Levey (1966), for instance, translated both *kankar* and *kankarzad* (كنكرزد) from Ibn Wahshiyya’s *Book of Poisons* as the artichoke. While uncertainty about terminology and plant names remains and we cannot make definitive claims as to the introduction of the artichoke at this time, it does however seem clear that different types of thistles were used for medicinal and food purposes in the southern Levant during the 10th c. ce.

Other vegetables that we hypothesize could have been incorporated into the southern Levantine crop curriculum during the 1st mill. ce, but that have thus far eluded archaeobotanical attestation, include lettuce, *Spinacia oleracea* (spinach), and *Corchorus* spp. (mallow, also referred to as jute mallow or *mulukhiyah*), though they are present in the written sources. Spinach is for instance cursorily mentioned in the 10th c. ce by Ibn Wahshiyya (Watson 1983; note 10, p 176 states that al-Nuwayri in the 14th c. ce mentions “a description of Ibn Wahshiyya’s recommendations for growing spinach” likely from the *Filaha Nabatiya*, though he did not come across it in the Cairo manuscript that he had access to when conducting his study). For jute mallow Löw (1967; Vol II, p 247; cf. Amar 2000, p 283) has noted that the term *melchiniki* from the *Jerusalem Talmud* is associated with the term *mulukhiya*, which is the Arabic term for both the plant and prepared foodstuff in Bilad al-Sham. In addition, in Ibn Wahshiyya’s *Book of Poisons* the leaves, twigs and roots of mallow are also mentioned as ingredients to make antidotes (Levey 1966). Levey specifies terms for mallow (خبازى), marshmallow (خطمى), marshmallow rose (likely referring specifically to the flower; ورد الخطمى) and two additional terms as potential names of different types of mallows (تودريك and سينتى); he does not mention *mulukhiya* (ملوخية) (*idem*, p 118, 121, 127). Lettuce is referred to in the *Mishna* (e.g. *Kilayim* 1:2) as *hazeret*. The earliest attestation of lettuce from the southern Levant comes from a letter (Papyrus No. 741) that was excavated at Masada and is dated between the start of construction of the fortress by Herod the Great in 37 bce and its destruction by the Romans in the spring of 73 ce. In this document, lettuce is referred to by the term *µαρούλιον* (*maroullin*) (Cotton and Geiger 1989, pp 85–88, Plate 8). Lettuce is widely mentioned in the works of Roman authors, such as Pliny and Columella (both 1st c. ce), and it also occurs as a food and medicinal plant in the Byzantine *Geoponika*. There *maroullin* is mentioned as both a general term for lettuce, as well as a variety of lettuce alongside others such as *thridakin* (Dalby 2011, pp 246, 252–253). Al-Muqaddasī mentions lettuce as a crop of Palestine (de Goeje 1877, p 181; Collins 2001, p 152). Levey notes that lettuce (الخسّ) in Ibn Wahshiyya’s *Book of Poisons* is associated with the term *thridax* (*hemeros*) from Dioscorides and Galen (Levey 1966; note 307).

### Oil, fibre and dye crops

The cultivation of *Linum usitatissimum* (linseed/flax), which can be used both for the production of linseed oil and linen, seems to already have started during the Neolithic, as attested through archaeobotanical evidence from Jericho (Hopf 1983). Its cultivation continues well through the IA, as attested at Deir Alla (van Zeist and Heeres 1973; Neef 1989) and presumably into the 1st mill. ce, after which *Gossypium arboretum*/*herbaceum* (Old World cotton) gradually became the primary source of plant-based fibre. Old World cotton was introduced into the region during the Roman period, as attested in the Classical sources and the *Mishna* (Decker 2009), and the earliest archaeobotanical evidence dates to the Late Roman period (ca. 106–324 ce) from Aila (Aqaba). At the same site, it was also found in early Byzantine, Umayyad, and Abbasid contexts (Ramsay and Parker 2016). In addition, cotton textiles were also identified at Ein Bokek dating to the Roman–Byzantine period (Sheffer and Tidhar 1991). Therefore, we have designated it as a RAD crop. Granger-Taylor hypothesized that around the Dead Sea cotton may have overtaken wool as the most important textile as early as the 7th c. ce (Granger-Taylor 2012). The cotton industry continued to expand during the Early Islamic period. This is supported by the large number of cotton textile finds from this region in that period relative to all other textile types, particularly at Nahal Omer (Shamir et al. 2023). Cotton textiles and candle wicks are attested from the 7th c. ce at Deir ‘Ain ‘Abata and cotton seeds were also found in 13th c. ce contexts at Khirbet Faris (Hoppé 1999; Granger-Taylor 2012; Hansen 2012). Cotton is also mentioned as a crop cultivated in Jerusalem in the 10th c. ce by al-Muqaddasī. Syrian cotton (i.e. from Bilad al-Sham) became very important in the textile trade and was highly sought after in (southern) Europe between the 13th and 16th centuries ce (cf. Mazzaoui 1981).

Water resources would have been very limited in some of the main areas in which cotton has been found, such as in the hinterland of Aila, the area around the Dead Sea (Deir ‘Ain ‘Abata), and the hinterland of Kerak (Khirbet Faris), especially during the growing season of a summer crop such as cotton. Its expansion alongside the cultivation of other summer crops, such as sesame, millets, and sorghum, is therefore likely linked to the development of more sophisticated water management and water storage and delivery systems between the Roman and Early Islamic periods (Lightfoot 1997, 2000; Abudanh and Twaissi 2010; Berenfield et al. 2016; Ortloff 2020; Avni 2021). During the ripening phase, the dry circumstances of these locales would have been a boon: cotton is a so-called ‘dry harvested crop’ that prior to the harvest is left to die and dry-out (Brouwer and Heibloem 1986). Cotton would therefore not compete with other summer crops over water resources when they were most scarce. Together these factors may have helped enable an agricultural specialisation in cotton production at rural sites in arid areas.

*Cannabis sativa* (hemp)/*Cannabis indica* (cannabis) are fibre crops of which the seeds can be pressed for oil or can be eaten as a porridge. They are associated with the Hebrew term *kanbos* (קַנְבּוֹס), which is directly related to the Greek term κανναβις and the Arabic term القنب. Hemp has been documented in the *Mishna* from the 2nd c. ce (e.g. *Kilayim* 9:1), where the term refers to the textile fabric rather than the plant itself. Amar and Iluz (2017) mention hemp as a new crop, the use of which expanded during or after the Early Islamic period. Later, in the 10th c. ce, al-Muqaddasī mentions that hemp fabrics (ثياب القنب) from al-‘Askar in the Khuzistan region are among the fabrics ‘the people of al-Ahwaz use’ and that the hemp cloth produced in the region of al-Daylam, the lowlands and highlands of Gilan (Iran), “is known in Egypt and al-Iraq”, implying that it was traded regionally (Collins 2001, pp 287, 340). This confirms that al-Muqaddasī was aware of areas specialising in hemp cultivation and the widespread use of its fabrics, but he does not provide information on whether or not it was cultivated and/or used for fabrics in Bilad al-Sham. When al-Muqaddasī deals with the region of Aqur (Al-Jazira), in Upper Mesopotamia, two terms are used in which both hemp and potentially cannabis are mentioned. For fabrics القنب is referenced as well as another word (الشاهدانق), which Collins translates as cannabis seed: “From Ma’lathaya, [come] dairy products, coal, grapes, fresh fruit, cannabis seed, hemp, and dried meat” (Collins 2001, p 123). الشاهدانق comes from the Middle Persian *šhdʾnk’* (or *šahdānag* or شَاهْدَانَج in Arabic) meaning literally ‘royal seed/grain’ and is associated with the hemp/cannabis seed. Hence, it is clear that the different plant parts were also commodities in their own right during this period. While consumers in the southern Levant would have been familiar with at least the fabrics, due to a lack of archaeobotanical remains and explicit written sources, we cannot pinpoint the exact moment of introduction of these crops into the region.

*Sesamum indicum* (sesame) cultivation, likely for both oil and seed production, is first attested archaeobotanically in the southern Levant from the IA at Deir Alla (Neef 1989). Columella (1st c. ce) notes that sesame is a summer crop and was cultivated in Syria (*De Re Rustica* 2.10.18; Bedigian 2004), while it is mentioned in the *Mishna* by the 2nd c. ce (Feliks 1980, p 129). It remained an important crop in the southern Levant and is mentioned both in Early Islamic and Late Medieval Arabic sources when, besides a foodstuff, it is also mentioned as a medicinal plant (Bedigian 2004). While explicit evidence from the southern Levant is currently lacking, we do however hypothesize that sesame could have been already introduced and cultivated sometime from the BA onwards. Both archaeobotanical evidence and written sources from Mesopotamia, Egypt, and the Gulf attest to its widespread cultivation and trade throughout southwest Asia during this period (Bedigian and Harlan 1986; Bedigian 2004; Zech-Matterne et al. 2015; Valera et al. 2022). Olive, discussed above in the section on fruit trees, would of course have served as the primary oil crop. Indeed, the earliest evidence for olive oil production was attested in the southern Levant (Galili et al. 1997).

Many plant dyes were developed in the ancient world, though one particular dye became very important for the southern Levant: indigo (Balfour-Paul 1997). It is referred to as *al-nīl* (النيل) in Early Islamic written sources, alluding to the River Nile, because of its brilliant blue hue. While today *Indigofera tinctoria* has become the most commonly cultivated *Indigofera* species, in the past other taxa within the genus were often used. As there currently is no evidence as to the introduction of *I. tinctoria* in the southern Levant, it is likely that other taxa were used here as well. Local, native taxa include *I. coerulea*, *I. articulata*, and *I. oblongifolia*, though only the first two taxa have been discussed as plants exploited for indigo production in the past. In the *Flora Palaestina* and in the modern checklist of plants of Jordan^3^, *I. articulata* is attested in multiple sub-regions (cf. Danin 2004). *I. coerulea* grows wild in the Dead Sea region and some have argued that it is the main native *Indigofera* sp. taxon throughout the region, challenging earlier identifications of *I. articulata* (Baumwol and Fragman-Sapir 2019). Archaeobotanically, it is difficult to distinguish between the two species’ seed morphology especially when the seeds are charred. The earliest archaeobotanical evidence of *I. articulata*/*coerulea* comes from the Byzantine site of Ein Gedi, where many seeds were found (6th c. ce; Melamed and Kislev 2005), and these have been associated with indigo production. Indigo seeds have been mentioned in the *Cairo Geniza* for their medicinal uses for skin diseases, wounds, and swellings as well, so it is possible that the archaeobotanical seeds may have resulted from more than one type of use (Lev and Amar 2008). Slightly later at Byzantine Zoara (early 7th c. ce), a fruit of *Indigofera* sp. was also encountered; this find likely originated from *Indigofera* sp. crop processing waste being used as a fuel source (Hansen 2023; Hansen and Heinrich in press). In his work on the flora, fauna, and agriculture in the *Mishna*, Feliks (1982) states that earlier exploitation of indigo dye is attested; he appears to be referring to *I. articulata*. While the *Mishna* was redacted in the 2nd c. ce, some of the source material has been traced back to the 1st c. bce. Feliks (1982) also suggested that Hasmonean palatial installations in Jericho (2nd–1st c. bce) were used for indigo processing. Amar and Iluz (2017) have suggested that similar evidence was found at a Herodian workshop in Ein Bokek (1st c. bce) where sediment analysis also indicated the presence of indigo compounds. Earlier evidence of the dye substance indigotin has been encountered on linen fragments from IA contexts at Kuntillat ‘Arjud. Indigo-dyed wool and linen fabrics became much more common during the Roman and Byzantine periods, though the specific plant source cannot be identified based on the chemical compound alone (Balfour-Paul 1997, p 6). Al-Muqaddasī confirms that indigo still was an important crop in Palestine, especially in the southern Ghors, into the 10th c. ce (de Goeje 1877, p 181; Collins 2001, pp 146, 152). Additional archaeological evidence for the cultivation and processing of indigo comes from Abbasid period contexts (9th–11th c. ce) at Khirbet ash-Sheikh ‘Isa (the Islamic settlement at Zoara) where many tools and vessels associated with these activities were excavated (LaGro 2002; Politis 2021).

### Condiments, herbs, medicinal plants, and sweeteners

A wide variety of plant taxa that can be used as herbs, spices, or condiments are native plants to the southern Levant that are well attested in the archaeobotanical record (cf. LBA Megiddo, Langgut et al. 2016) as well as in the written sources (cf. Amar 2000; Collins 2001; Amar and Lev 2018). These plants would locally have been widely available; examples include coriander, *Salvia officinalis* (sage), mint, *Myrtus communis* (myrtle) and *Thymus* sp. (thyme). Coriander seeds were archaeobotanically attested at IA Deir Alla and were likely cultivated at or nearby the site (Neef 1989). Many plants in this category were also held to possess medicinal properties, for which there is ample evidence in the Classical sources and Early and Middle Islamic sources (Amar 2002; Lev 2002; Amar and Lev 2018 for plant lists and respective list of sources). For the purpose of this paper, however, we would like to highlight only three taxa for which we have both evidence for their introduction as well as their cultivation: *Cuminum cyminum* (cumin), *Nigella sativa* (black cumin), and *Papaver somniferum* (opium poppy). Though already attested in BA (Assyrian) written sources, where it was mentioned for its medicinal purposes, the earliest archaeobotanical evidence for black cumin from the southern Levant dates to the IA at Deir Alla (Neef 1989; Heiss et al. 2013). Later it was also found in Early Islamic (mid-8th–10th c. ce) Givati (Amichay and Weiss 2020). Classical and Early Islamic sources mention black cumin as a medicinal plant (e.g. Dioscordes’ *De materia medica*, Al-Kindi’s *De Gradibus*, and Al-Biruni’s *Book on the Pharmacopoeia of Medicine*), while later medieval sources also mention its use as a condiment (see Amichay and Weiss 2020 for a detailed review). Cumin appears to be another IA introduction and is attested at both Aphek and Deir Alla (Neef 1989; Frumin et al. 2015). Yet another crop that could be potentially used as a condiment, medicine, or drug, opium poppy, also seems to have been introduced during the IA, as attested by its earliest finds at Ashkelon (Mahler-Slasky 2004; Frumin et al. 2015). *Papaver* sp., identified only to the genus level, was found in LBA Megiddo, and it has been suggested that it was used for medicinal purposes (Langgut et al. 2016). Thus far, we did not encounter explicit archaeobotanical or written source evidence for specific condiments and herbs being introduced to the region during the 1st mill. ce.

Dates and honey were initially the main sugar-based foods, food preservatives, and sweeteners in the southern Levant, and there is evidence of sophisticated, industrial scale production of honey and beeswax dating to the IA in Tel Rehov in the Jordan Valley (Bloch et al. 2010). While honey remained an important product, in the 1st mill. ce, *Saccharum* sp. (sugarcane), a plant-based source of sugar, became available for manufacturing a variety of sugar products including molasses, cane sugar, sugar loaves and white sugar. Knowledge of the sugarcane plant entered the Classical sources from the Hellenistic period onwards, with it consistently being described as a type of reed that “produced honey without bees” that was to be found in India and Yemen (Sato 2015, pp 16–17). The crop itself however was only introduced into Egypt and Syria later, alongside its processing technology and cultivation knowledge (cf. Gopal 1964). Evidence from written sources places the earliest sugacane cultivation and production in Egypt in the 8th c. ce (Watson 1983; Mikhail 2000). In the 10th c. ce, it is referenced as being produced in different areas in the southern Levant and is praised for its quality by al-Muqaddasī (Collins 2001, pp 151–152). With the help of new innovations, the sugar industry would continue to thrive during Ayyubid and Mamluk periods, well into the 2nd mill. ce (Sato 2004, 2015; Politis 2013a, 2015, 2021). Archaeologically the trade of sugar is attested through the ceramic ‘sugar pots’ that were encountered throughout the region in the Mamluk period, for instance at Tall Hisban and Tall Abu Sarbut (Walker 2004, 2011; Steiner 2008). Moreover, the production of sugar in the region is attested through 43 sugar factories that were identified and surveyed thus far in the Transjordan and Palestine, including those at Tawahin as-Sukkar, Jericho, and in the ‘Akko plain dating from the Fatimid through the early Ottoman period, when the industry began to decline under the influence of European competition (Hamarneh 1978; Abu Dalu 1995; Stern 1999; Taha 2004, 2009, 2015; Abu-dalo 2010, 2015; Politis 2013a, b, 2015, 2021; Stern et al. 2015). The first archaeobotanical evidence, however, for desiccated sugarcane dates to 11th–13th c. ce contexts (more precisely dated to 1120–1210 cal ce) in Quseir al-Qadim, Egypt (van der Veen 2011). In the southern Levant, the first archaeobotanical evidence of sugarcane is attested in Abbasid/Fatimid (8th–11th c. ce) and Ayyubid/Mamluk (12th–15th c. ce) contexts in the town of Khirbet ash-Sheikh ‘Isa and its sugar factory, the Tawahin as-Sukkar. This material is charred and consists of culms, lower culms, and rhizomes, and attests to the use of sugarcane pressing remains as a fuel source called bagasse (Hansen 2023; Hansen and Heinrich in press).

## Discussion

**Fig. 2:**
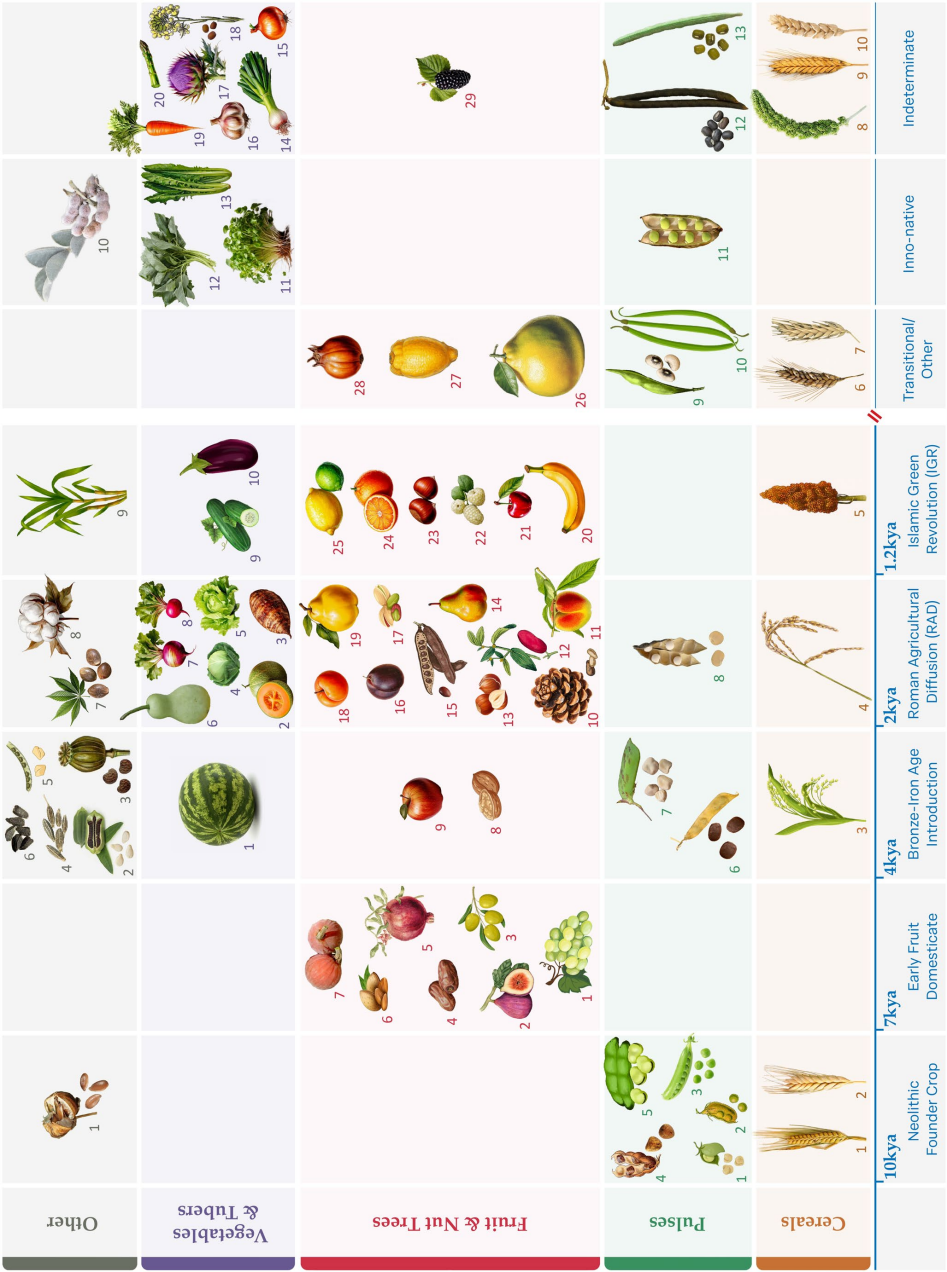
Crop types and taxa in the southern Levant by earliest attestation. Cereals: *1* Two-row barley; *2* Emmer wheat; *3* Broomcorn millet; *4* Rice; *5* Sorghum; *6* Free threshing tetraploid wheat; *7* Six-row barley; *8* Foxtail millet; *9* Einkorn wheat; *10* Free threshing hexaploid wheat. *Pulses*: *1* Chickpea; *2* Lentil; *3* Common pea; *4* Bitter vetch; *5* Broad bean; *6* Spanish vetchling; *7* Grass pea; *8* White lupine; *9* Cowpea; *10* Yardlong bean; *11* Cyprus vetch; *12* Black gram; *13* Mung bean. Fruit and nut trees: *1* Grape; *2* Fig; *3* Olive; *4* Date; *5* Pomegranate; *6* Almond; *7* Sycamore fig; *8* Persian walnut; *9* Apple; *10* Pine nut; *11* Peach; *12* Chinese date/True jujube; *13* Hazelnut; *14* Pear; *15* Carob; *16* Plum; *17* Pistachio nut; *18* Apricot; *19* Quince; *20* Banana/plantain; *21* Cherry; *22* White mulberry; *23* Chestnut; *24* Bitter orange; *25* Lemon/lime; *26* Pomelo; *27* Citron; *28* Medlar. 29. Black mulberry. Vegetables and tubers: *1* Watermelon; *2* Musk melon; *3* Taro; *4* Cabbage; *5* Lettuce; *6* Bottle gourd; *7* Turnip; *8* Radish; *9* Cucumber; *10* Aubergine; *11* Garden cress; *12* Jute/mallow/mulukhiyah; *13* Chicory/endive; *14* Leek/shallot; *15* Onion; *16* Garlic; *17* Cardoon/artichoke thistle; *18* Black mustard; *19* Carrot; *20* Asparagus. Other: *1* Flax/linseed; *2* Sesame; *3* Opium poppy; *4* Cumin; *5* Fenugreek; *6* Black cumin; *7* Hemp/cannabis; *8* Cotton; *9* Sugarcane; *10* Indigo (Design by Sapir Haad)

In this review we assessed and synthesized the available archaeobotanical and historical evidence pertaining to the availability and introduction of new crops (both through diffusion and Inno-native Crop/Food Plant exploitation) in the southern Levant, paying particular attention to the 1st mill. ce. While there are still some gaps in our data both from the archaeobotanical record and written sources as to the moment of diffusion of certain crops (e.g. the introduction of spinach, free-threshing naked hexaploid wheat, and asparagus), and as to the question when certain local, native plants began to be actively cultivated (e.g. carrot, leek/shallot, onion, garlic, black mustard, chicory, and Syrian pear), various trends emerge from the available data. The complete visual representation of crop types and taxa in the southern Levant by their earliest attestation is shown in Fig. 2.

Between the Neolithic and EBA, the basis of the crop curriculum was formed with the exploitation of cereals and pulses as staples, which were later supplemented by cultivated fruit and nut trees. During these and subsequent periods, the southern Levantine crop basket was primarily composed of crops native to West Asia and the Eastern Mediterranean. These were complemented by Inno-native Crops/Food Plants—local/native plants from the southern Levant that had already been previously present in the environment, but that would start to be managed and cultivated after the onset of agriculture (e.g. coriander) (cf. Frumin et al. 2015). More tree crops, vegetables, and condiments were gradually added to the crop curriculum from the MBA to the Hellenistic period, such as black mulberry and grass pea, further diversifying the range of available crops in the 1st mill. bce. While the use of some of these crops would diminish, most would continue to be constants within the crop curriculum of the southern Levant.

With respect to the ‘long 1st mill. ce’, two main periods of diffusion can be identified. The first of these centers on the Roman period, with possible late Hellenistic contributions (Heinrich 2017; Fuks et al. 2023). This period of crop introductions (1st c. bce–4th c. ce) is labelled the “Roman Agricultural Diffusion” (RAD) and includes white lupine, improved carob cultivars, hazelnut, quince, pine nut, pistachio nut, apricot, plum, peach, pear, Chinese date, taro, cabbage, turnip, musk melon, lettuce, radish, and hemp/cannabis. The additions of the fruit and nut trees above constitute a key component of the Roman Agricultural Diffusion (RAD) phenomenon. The Romans were experts in fruit tree improvement, including grafting techniques that increased fruit yield and quality (cf. Marzano 2022, pp 130–176); it is possible that the diffusion of this knowledge stimulated the adoption of new fruit trees in areas where they previously were not cultivated (cf. Amichay and Weiss 2020). Indigo appears to have been produced from native plants in the *Indigofera* genus that started being exploited as Inno-native Crops/Food Plants in the Byzantine period. Some of the pre-Islamic introductions, such as hard wheat, rice, cotton, and watermelon, were previously held by Watson to have been Early Islamic introductions. Notwithstanding, from the evidence available thus far the Early Islamic period (mid-7th to 12th c. ce) does signify a second intense episode of diffusion during the 1st mill. ce. Many of the IGR crop introductions that had been proposed by Watson can be confirmed based on current evidence such as banana/plantain, bitter orange, aubergine, and sugarcane. In addition, various other crops not discussed by Watson, such as chestnut, white mulberry, cherry, and cucumber are first attested or mentioned in the southern Levant in the Early Islamic period (Amar 2000).

**Fig. 3:**
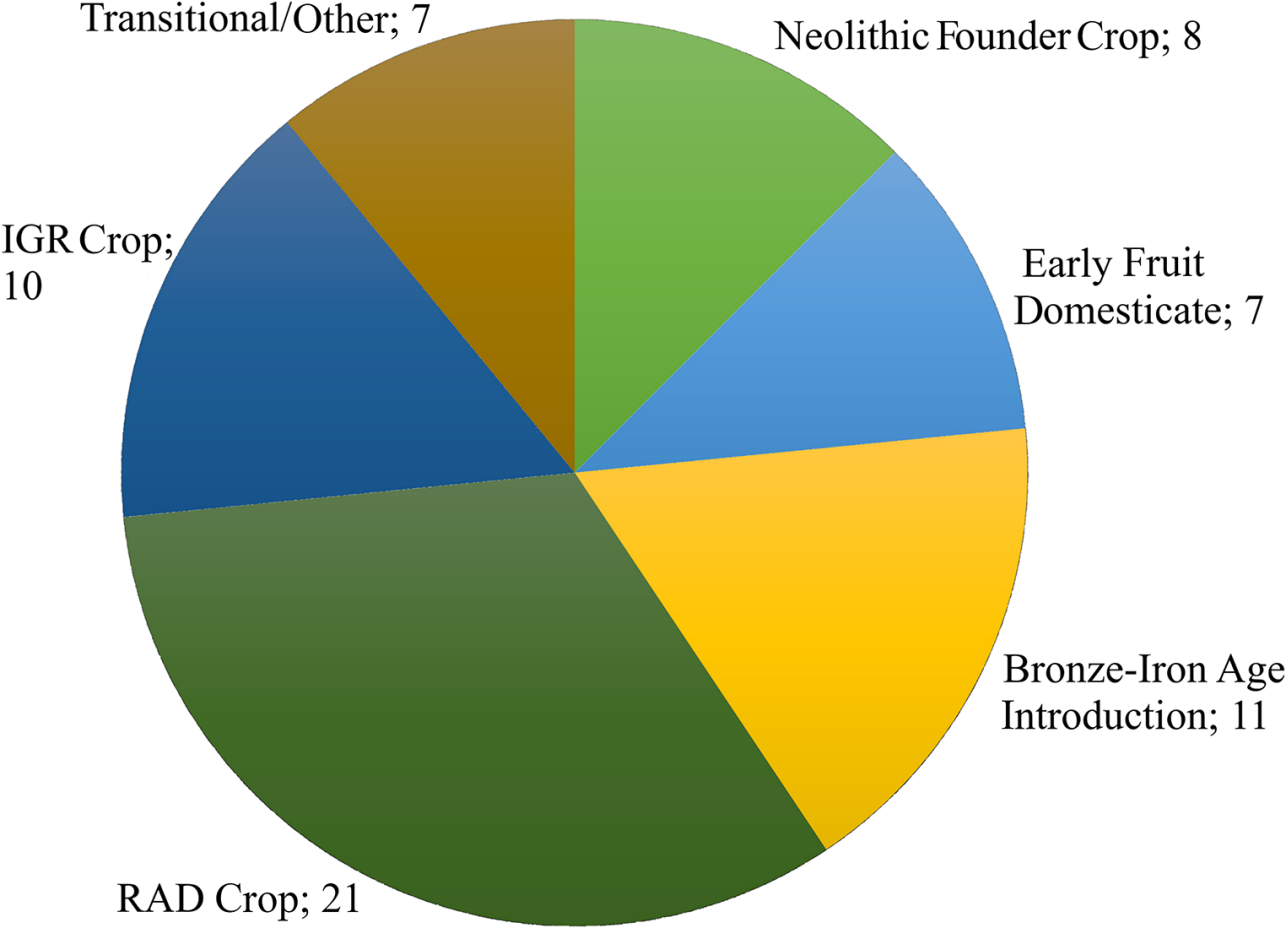
Chronology of earliest attestation of crop taxa in the southern Levant arranged by crop tag (*n* = 64). Introductions with an indeterminate date (*n* = 11) and Inno-native Crops/Food Plants (*n* = 5) are excluded from the pie chart

Together, the numbers of both RAD and IGR crop introductions are very high in comparison to other periods of the last 10,000 years. 1st millennium introductions account for nearly half of those on our list (Fig. 3), demonstrating that more crops were introduced to the southern Levant in the 1st mill. ce than ever before. This is especially so when considering that the RAD and the IGR cover the shortest chronological periods in our analysis and are periods that have archaeologically and archaeobotanically been less intensively studied. On the other hand, they are also the periods best represented in our written sources. These factors represent opposite biases, as we outlined above in our methodological considerations; caution is therefore needed in drawing conclusions from comparing more distant (pre-/proto-historical) and more recent (historical) periods. A significant result of this review nonetheless is that the RAD crops in our database (*n* = 21) outnumber IGR crops (*n* = 10) by a factor of two (Fig. 3). The difference between the two phases is even greater when considering the archaeobotanical data alone: among the IGR crops only aubergine and sugarcane were attested in the archaeobotanical record of the southern Levant as opposed to many RAD crops (e.g. white lupine, cowpea, hazelnut, pine nut, pistachio, plum, and peach). This latter phenomenon may be partially explained by taphonomic bias: many of the IGR crops are tropical vegetables used for their leaves, roots, or unripe fruits, which are much less likely to leave traces in the archaeobotanical record than the fruit stones and seeds that typify the above RAD crops. Indeed, the two IGR crops found so far were discovered under conditions of exceptional archaeological preservation. These biases demonstrate the importance of integrating both archaeobotanical and textual sources when assessing crop introductions, as well as the need for more Roman, Byzantine, and Islamic archaeobotany in the southern Levant.

Multiple conclusions may be drawn from these results. The smaller, and now reduced, number of IGR introductions (as compared to RAD introductions), challenges Watson’s thesis (1974, 1981, 1983) of the Early Islamic crop diffusion being as revolutionary as he proposed. We do not argue that this negates the Islamic Green Revolution: our data still show a significant number of introductions, some of which Watson did not (and because of the state of the field and data available at that time could not) incorporate into his model. It is moreover beyond the scope of the current paper to assess if the difference in the number of introduced taxa translates into a commensurate difference in economic impact—but as we build on the groundwork that Watson laid, it does require a reframing of the argument. In addition, one could argue that the introduction of the RAD crops, at least for our case study of the southern Levant, represents an agricultural revolution in its own right. This appears to have been fuelled by increased market integration, connectivity, and proto-globalization. These are developments that can be observed in much of the Mediterranean from the 1st mill. bce onwards (cf. Lentjes 2016), and which culminated under the Roman unification of the region and the subsequent *Pax Romana* (e.g. Heinrich 2017). It could subsequently be argued that Watson’s Early Islamic crop diffusion continued the same long-term trend of increased connectivity and the exchange of crops and ideas, but of a different origin: the IGR crops reflect connectivity beyond the Mediterranean as the southern Levant became part of different Muslim Empires with other geographic orientations.

After the 10th c. ce, the introduction of new crops appears to slow down, though new taxa occasionally still appear, such as the inferred Late Medieval introduction of pomelo during the 13th c. ce. A new period of intense diffusion such as seen during the 1st mill. ce would not be witnessed until the second half of the 2nd mill. ce with the introduction of crops from the Americas. This does not mean that the periods in between these episodes of intense diffusion were stagnant. In fact, following intense episodes of diffusion, there appear to have been periods of consolidation during which the new crops were integrated into the agricultural regime and the effects of the introduction were more widely felt (cf. van der Veen 2010). Cotton may have been a Roman introduction, but it seems to have become a dominant fibre crop from the Byzantine and Early Islamic periods onwards, and the cotton industry remained important long after. Indigo appears to be a Byzantine innovation, but it is during the Abbasid period that the industry boomed and gained renown, and while sugarcane is attested in the Levant from the 10th c. ce on, the heyday of the Levantine sugarcane industry is situated in the Crusader, Ayyubid, and Mamluk periods (12th–15th c. ce). As such the diffusion of crops into the Levant was a sign of the region’s integration into larger political units and its connectivity with other regions. It also sowed the seeds for the development of specialised agricultural industries that would in turn shape its global commercial and economic interactions in subsequent centuries.

## Supplementary Information

Below is the link to the electronic supplementary material.

Supplementary Material 1

## Footnotes

1 We would like to note that for the southern Levant wild einkorn (*Triticum monococcum* ssp. *baeoticum*) has been attested at Natufian Shubayqa 1 in the processing and preparation of ‘bread-like products’. The pre-agriculture gathering of wild emmer (*Triticum turgidum* ssp. *dicoccoides*) and wild barley (*Hordeum vulgare* ssp. *spontaneum*) has been attested at early Epi-Palaeolithic Ohalo II; wild emmer has also been attested at PPNA Netiv Hagdud and Gilgal (Arranz-Otaegui et al. 2018; see Zohary et al. 2012, p 41 and case studies therein). However, an in-depth discussion of the spread/use of wild wheats and barleys in the southern Levant is beyond the scope of this paper; we here refer the reader to the discussion in Zohary et al. 2012, pp 34–47, 51–59.

2 Some publications’ identifications refer to *Lathyrus sativus*/*cicera*, while others refer to *Lathyrus sativus*. We have chosen to combine *Lathyrus sativus/cicera* into the lowest common denominator category for this crop in our ESM. It has been argued that the two species are indistinguishable in charred seeds. For a discussion on *Lathyrus* consumption in the southern Levant, see Mahler-Slasky and Kislev 2010.

3 “Checklist of Plants of the Hashemite Kingdom of Jordan - ODU Plant Site”. n.d. https://ww2.odu.edu/~lmusselm/plant/jordan/.

## References

[CR1] Abbo S, Gopher A, Lev-Yadun S (2015) Fruit domestication in the Near East. Plant Breed Rev 39:325–378. 10.1002/9781119107743.ch7

[CR3] Abu Dalu R (1995) The technology of sugar mills in the Jordan Valley during the Islamic periods. Stud Hist Archaeol Jordan 5:37–48 (in Arabic)

[CR2] Abu-dalo R (2010) Sugar mills (Tawahin es-Sukkar) in the Jordan Valley. Int Molinol 80:26–34

[CR4] Abudanh F, Twaissi S (2010) Innovation or technology immigration? The Qanat systems in the regions of Udhruh and Ma’an in Southern Jordan. Bull Am Sch Orient Res (BASOR) 360:67–87. 10.1086/BASOR41104419

[CR5] Amar Z (2000) Agricultural produce in the land of Israel in the Middle Ages. Yad Yitzhak Ben-Zvi, Jerusalem. (in Hebrew)

[CR6] Amar Z (2002) The history of medicine in Jerusalem. BAR International Series 1032. BAR Publishing, Oxford

[CR7] Amar Z (2012) Flora of the Bible. Rubin Mass Ltd., Jerusalem

[CR8] Amar Z, Iluz D (2017) Balsam: the most expensive perfume plant in the Ancient World. In: Ferziger A, Sperber D (eds) The paths of Daniel: studies in Judaism and Jewish culture in honor of Rabbi professor Daniel Sperber. Bar-Ilan University, Ramat-Gan, pp 15–27

[CR9] Amar Z, Lev E (2011) Watermelon, Chate melon and cucumber: new light on traditional and innovative field crops of the Middle Ages. J Asiat 299:193–204. 10.2143/JA.299.1.2131063

[CR10] Amar Z, Lev E (2018) Arabian drugs in Early Medieval Mediterranean medicine. Edinburgh University, Edinburgh

[CR12] Amichay O, Weiss E (2020) The archaeobotanical remains. In: Ben-Ami D, Tchekhanovets Y (eds) Jerusalem excavations in the Tyropoeon Valley (Givati Parking Lot), vol 2: the Byzantine and Early Islamic Periods: Part 2:Strata IV/I. The Early Islamic Period. IAA Reports 66/2. Israel Antiquities Authority, Jerusalem, pp 645–701

[CR11] Amichay O, Ben-Ami D, Tchekhanovets Y, Shahack-Gross R, Fuks D, Weiss E (2019) A bazaar assemblage: reconstructing consumption, production and trade from mineralised seeds in Abbasid Jerusalem. Antiquity 93:199–217. 10.15184/aqy.2018.180

[CR13] Aouizerat T, Gutman I, Paz Y et al (2019) Isolation and characterization of live yeast cells from ancient vessels as a tool in bio-archaeology. mBio 10:e00388. 10.1128/mbio.00388-19 31040238 10.1128/mBio.00388-19 PMC6495373

[CR14] Arranz-Otaegui A, Gonzalez Carretero L, Ramsey MN, Fuller DQ, Richter T (2018) Archaeobotanical evidence reveals the origins of bread 14,400 years ago in Northeastern Jordan. Proc Natl Acad Sci USA 115:7925–7930. 10.1073/pnas.1801071115 30012614 10.1073/pnas.1801071115 PMC6077754

[CR15] Asadi-Samani M, Rafieian-Kopaei M, Azimi N (2013) Gundelia: a systematic review of medicinal and molecular perspective. Pak J Biol Sci 16:21:1238–1247. 10.3923/pjbs.2013.1238.1247 24511731 10.3923/pjbs.2013.1238.1247

[CR16] Asouti E, Kabukcu C, White CE, Kuijt I, Finlayson B, Makarewicz C (2015) Early Holocene woodland vegetation and human impacts in the arid zone of the Southern Levant. Holocene 25:1565–1580. 10.1177/0959683615580199

[CR17] Avital A, Paris HS (2014) Cucurbits depicted in Byzantine mosaics from Israel, 350–600 CE. Ann Bot 114:203–222. 10.1093/aob/mcu106 24948671 10.1093/aob/mcu106 PMC4111391

[CR18] Avni G (2021) Terraced fields, irrigation systems and agricultural production in Early Islamic Palestine and Jordan: continuity and innovation. J Islam Archaeol 7:111–137. 10.1558/jia.17679

[CR19] Avni Y, Avni G, Porat N (2019) A review of the rise and fall of ancient desert runoff agriculture in the Negev Highlands – a model for the Southern Levant deserts. J Arid Environ 163:127–137. 10.1016/j.jaridenv.2019.01.010

[CR20] Balfour-Paul J (1997) Indigo in the Arab world. Routledge, London

[CR21] Barreiro PG (2019) Akkoub: the wild and thorny Eastern Mediterranean secret. Kew Gardens, April 4, 2019. https://www.kew.org/read-and-watch/akkoub-wild-thorny-Mediterranean-secret

[CR22] Baruch U (2007) Identification of wood remains from Area R. In: Mazar A, Mullins RA (eds) Excavations at Tel Beth-Shean 1989–1996, vol 2: the Middle and late Bronze Age strata in Area R. the Beth-Shean Valley archaeological project publication 2. The Hebrew University of Jerusalem, Jerusalem, pp 716–717

[CR23] Batlle I, Tous J (1997) Carob tree: *Ceratonia siliqua* L. Promoting the conservation and use of underutilized and neglected crops 17. Institute of Plant Genetics and Crop Plant Research/International Plant Genetic Resources Institute, Gatersleben/Rome

[CR24] Baumwol Z, Fragman-Sapir O (2019) *Indigofera coerulea* Roxb. In: Danin A, Fragman-Sapir O (eds) Flora of Israel and adjacent areas. Jerusalem Botanical Gardens. https://flora.org.il/en/plants/indart/

[CR25] Bedigian D (2004) History and Lore of sesame in Southwest Asia. Econ Bot 58:329–353. https://www.jstor.org/stable/4256831

[CR26] Bedigian D, Harlan JR (1986) Evidence for cultivation of sesame in the ancient world. Econ Bot 40:137–154. 10.1007/BF02859136

[CR27] Berenfield ML, Dufton JA, Rojas F (2016) Green Petra: archaeological explorations in the city’s Northern wadis. Levant 48:79–107. 10.1080/00758914.2015.1107299

[CR28] Besseiche M, Chambraud E, Dabrowski V, Brandstatt E, Sabot F, Bouchaud C, Gros-Balthazard M (2025) DateBack, an evolving open-access repository of Phoenix archaeobotanical data supporting new perspectives on the history of date palm cultivation. Peer Community J 5:e55. 10.24072/pci.archaeo.100603

[CR29] Bloch GT, Francoy TM, Wachtel I, Panitz-Cohen N, Fuchs S, Mazar A (2010) Industrial apiculture in the Jordan Valley during biblical times with Anatolian honeybees. Proc Natl Acad Sci USA 107:11240–11244. 10.1073/pnas.1003265107 20534519 10.1073/pnas.1003265107 PMC2895135

[CR30] Bouchaud C, Jacquat C, Martinoli D (2017) Landscape use and fruit cultivation in Petra (Jordan) from Nabataean to Byzantine times (2nd century BC–5th century AD). Veget Hist Archaeobot 26:223–244. 10.1007/s00334-016-0582-y

[CR31] Brouwer C, Heibloem M (1986) Chap. 2: crop water needs. In: Brouwer C, Heibloem M (eds) Irrigation water management. Training manual 3. Irrigation water needs. FAO, Rome. http://www.fao.org/docrep/s2022e/s2022e02.htm

[CR32] Cappers RTJ (2006) Roman foodprints at Berenike: archaeobotanical evidence of subsistence and trade in the Eastern Desert of Egypt. Cotsen Institute of Archaeology, University of California, Los Angeles

[CR34] Caracuta V, Weinstein-Evron M, Kaufman D, Yeshurun R, Silvent J, Boaretto E (2016) 14,000-year-old seeds indicate the Levantine origin of the lost progenitor of faba bean. Sci Rep 6:37399. 10.1038/srep37399 27876767 10.1038/srep37399 PMC5120295

[CR33] Caracuta V, Vardi J, Paz Y, Boaretto E (2017) Farming legumes in the Pre-Pottery Neolithic: new discoveries from the site of Ahihud (Israel). PLoS ONE 12:e0177859. 10.1371/journal.pone.0177859 28542358 10.1371/journal.pone.0177859 PMC5443508

[CR35] Cartwright CR (1997) Interim Report on the archaeobotanical remains from the 1996 season of excavations of the Early Bronze Age complex at Tell es-Sa’idiyeh, Jordan. Palest Explor Q 129:72–75

[CR36] Cartwright CR, Clapham A (1993) The archaeobotanical remains from Tell es-Sa’idiyeh, Jordan. Palest Explor Q 125:73–74

[CR37] Caspi B, Kislev ME, Simchoni O, Melamed Y (2020) Food-Plant remains. In: Mazar A, Panitz-Cohen N (eds) Tel Reḥov: A Bronze and Iron Age city in the Beth-Shean Valley. Various objects and Natural-Science Studies. Qedem, vol 5. The Institute of Archaeology, Hebrew University of Jerusalem, Jerusalem, pp 655–666

[CR38] Cohen P, Bacilieri R, Ramos-Madrigal J et al (2023) Ancient DNA from a lost Negev Highlands desert grape reveals a Late Antiquity wine lineage. Proc Natl Acad Sci USA 120:e2213563120. 10.1073/pnas.2213563120 37068234 10.1073/pnas.2213563120 PMC10151551

[CR39] Collins BA (Transl) (2001) The best divisions for knowledge of the regions (Aḥsan al-Taqāsīm fī Ma’rifat al-Aqālīm by al-Muqaddasī). Garnet Publishing, Reading

[CR40] Cotton HM, Geiger J (1989) No. 741: Letter of Abaskantos to Judas. In: Cotton HM, Geiger J (eds) Masada II. The Yigael Yadin excavations, 1963–1965. Final reports. The Latin and Greek documents. Israel Exploration Society. The Hebrew University of Jerusalem, Jerusalem, pp 85–88 and Plate 8

[CR42] Dalby A (2011) Geoponika farm work: a modern translation of the Roman and Byzantine farming handbook. Prospect Books, Blackawton

[CR41] Danin A (2004) Distribution atlas of plants in the Flora Palaestina area. The Israel Academy of Sciences and Humanities, Jerusalem

[CR44] De Goeje MJ (ed) (1877) Aḥsan al-Taqāsīm fī Ma’rifat al-Aqālīm by al-Muqaddasī (The best divisions for knowledge of the regions): descriptio imperii Moslemici / Auctore Schamso ‘d-din Abu abdollah Mohammed Ibn Ahmed Ibn abi Bekr al-Banna al-Basschari al-Mokaddasi. Brill, Leiden. (in Arabic)

[CR43] Decker M (2009) Plants and progress: rethinking the Islamic Agricultural Revolution. J World Hist 20:187–206. https://www.jstor.org/stable/40542757

[CR45] Farahani A (2018) A 2500-year historical ecology of agricultural production under empire in Dhiban, Jordan. J Anthropol Archaeol 52:137–155. 10.1016/j.jaa.2018.09.006

[CR46] Farahani A, Porter BW, Huynh H, Routledge B (2016) Crop storage and animal husbandry at Early Iron Age Khirbat al-Mudayna al-‘Aliya (Jordan): a paleoethnobotanical approach. In: McGeough KM (ed) The archaeology of agro-pastoralist economies in Jordan. American Schools of Oriental Research, Boston, pp 27–89

[CR47] Feinbrun-Dothan N (1978) Flora Palaestina. Part Three: Ericaceae to Compositae, 2 vols: text and plates. The Israel Academy of Sciences and Humanities, Jerusalem

[CR48] Feinbrun-Dothan N (1986) Flora Palaestina. Part Four: Alismataceae to Orchidaceae, 2 vols: text and plates. The Israel Academy of Sciences and Humanities, Jerusalem

[CR49] Feliks Y (1968) Plant world of the Bible. Masada Ltd., Ramat-Gan. In: Feliks Y (1980) The Jerusalem Talmud (Talmud Yerushalmi): Tractate Shevi’it. Zurot Press, Jerusalem, pp 128–129. (in Hebrew)

[CR50] Feliks Y (1982) The flora and fauna in the Mishnah: accompanied by a chapter on the tools of agriculture and its guidelines. המכון לחקר המשנה, Jerusalem. (in Hebrew)

[CR51] Feliks Y (1994) Fruit trees in the Bible and Talmudic literature. Rubin Mass, Jerusalem

[CR52] Feliks Y (2005) The Jerusalem Talmud, Tractate Ma’asrot: annotated critical edition. Bar-Ilan University, Ramat-Gan. (in Hebrew)

[CR53] Finné M, Holmgren K, Sundqvist HS, Weiberg E, Lindblom M (2011) Climate in the Eastern Mediterranean, and adjacent regions, during the past 6000 years – a review. J Archaeol Sci 38:3:153–3173. 10.1016/j.jas.2011.05.007

[CR54] Forste KM, Marston JM, Hoffman T (2022) Urban agricultural economy of the Early Islamic Southern Levant: a case study of Ashkelon. Veget Hist Archaeobot 31:623–642. 10.1007/s00334-022-00892-z

[CR55] Frayn JM (1979) Subsistence farming in Roman Italy. Centaur, London

[CR56] Frumin S, Maeir A, Kolska Horwitz L, Weiss E (2015) Studying ancient anthropogenic impacts on current floral biodiversity in the Southern Levant as reflected by the Philistine migration. Sci Rep 5:13308. 10.1038/srep13308 26304818 10.1038/srep13308 PMC4642518

[CR57] Frumin SI, Melamed Y, Weiss E (2019) The wheat-people of Canaan. In: Maeir AM, Shai I, McKinny C (eds) The Late Bronze and Early Iron Ages of Southern Canaan. De Gruyter, Berlin, pp 19–36

[CR58] Fuks D, Amichay O, Weiss E (2020a) Innovation or preservation? Abbasid aubergines, archaeobotany, and the Islamic Green Revolution. Archaeol Anthropol Sci 12:50. 10.1007/s12520-019-00959-5

[CR59] Fuks D, Bar-Oz G, Tepper Y, Erickson-Gini T, Langgut D, Weissbrod L, Weiss E (2020b) The rise and fall of viticulture in the Late Antique Negev Highlands reconstructed from archaeobotanical and ceramic data. Proc Natl Acad Sci USA 117:19780–19791. 10.1073/pnas.1922200117 32719145 10.1073/pnas.1922200117 PMC7443973

[CR60] Fuks D, Melamed Y, Langgut D, Erickson-Gini T, Tepper Y, Bar-Oz G, Weiss E (2023) Unprecedented yet gradual nature of first millennium CE intercontinental crop plant dispersal revealed in ancient Negev Desert refuse. eLife 12:e85118. 10.7554/eLife.85118 38011372 10.7554/eLife.85118 PMC10846859

[CR61] Fuller DQ, Stevens CJ (2019) Between domestication and civilization: the role of agriculture and arboriculture in the emergence of the first urban societies. Veget Hist Archaeobot 28:263–282. 10.1007/s00334-019-00727-4 10.1007/s00334-019-00727-4 PMC6499764 31118541

[CR62] Fuller DQ, Willcox G, Allaby RG (2011) Cultivation and domestication had multiple origins: arguments against the core area hypothesis for the origins of agriculture in the Near East. World Archaeol 43:628–652. 10.1080/00438243.2011.624747

[CR63] Galili E, Stanley DJ, Sharvit J, Weinstein-Evron M (1997) Evidence for earliest olive-oil production in submerged settlements off the Carmel Coast, Israel. J Archaeol Sci 24:1:141–1150. 10.1006/jasc.1997.0193

[CR64] Gopal L (1964) Sugar-making in Ancient India. J Econ Soc Hist Orient 7:57–72. 10.1163/156852064X00030

[CR65] Grabois A (1984) From ‘holy geography’ to ‘palestinography’: changes in the descriptions of Thirteenth Century pilgrims. Cathedra 31:43–66. https://www.jstor.org/stable/23398958

[CR66] Granger-Taylor H (2012) Chapter V.10d Miscellaneous objects: The textiles. In: Politis KD (ed) Sanctuary of Lot at Deir ‘Ain ‘Abata in Jordan: excavations 1988–2003. Jordan Distribution Agency in association with the British Museum, Amman, pp 378–391

[CR67] Gregg MW, Banning EB, Gibbs K, Slater GF (2009) Subsistence practices and pottery use in Neolithic Jordan: molecular and isotopic evidence. J Archaeol Sci 36:937–946. 10.1016/j.jas.2008.09.009

[CR68] Grimaldi IM, Muthukumaran S, Tozzi G, Nastasi A, Boivin N, Matthews PJ, van Andel T (2018) Literary evidence for taro in the ancient Mediterranean: a chronology of names and uses in a multilingual world. PLoS ONE 13:e0198333. 10.1371/journal.pone.0198333 29870533 10.1371/journal.pone.0198333 PMC5988270

[CR69] Grootveld E (2008) Archaeo-botanical report of the excavations of Tell Abu Sarbut. In: Steiner ML, van der Steen EJ (eds) Sacred and sweet: studies on the material culture of Tell Deir ‘Alla and Tell Abu Sarbut. Peeters, Leuven, pp 197–210

[CR70] Halabi S, Battah AA, Aburjai T, Hudaib M (2005) Phytochemical and antiplatelet investigation of *Gundelia tournifortii*. Pharm Biol 43:6496–6500. 10.1080/13880200500220268

[CR71] Hamarneh S (1978) Sugarcane cultivation and refining under Arab Muslims during the Middle Ages. Ann Depart Antiquities Jordan (ADAJ) 22:12–19 (in Arabic)

[CR72] Hansen AM (2012) Evaluating archaeobotanical material from Khirbet Faris: a comparative analysis and review of archaeobotanical data from early to medieval Islamic sites in the Levant and Africa. MSc Dissertation, Research Laboratory for Archaeology and the History of Art, University of Oxford, Oxford

[CR73] Hansen AM (2023) Preliminary results for archaeobotanical samples from Khirbat ash-Sheikh ‘Isa. In: Politis KD (ed) Ancient landscapes of Zoara II. Finds from surveys and excavations at the Ghor as-Safi in Jordan, 1997–2018. Routledge, London, pp 295–308

[CR74] Hansen AM (in press) Chap. 7.2: Land use and foodways: the macrobotanical record. In: Walker BJ (ed) Life on the farm in Late Medieval Jerusalem: the village of Beit Mazmil, its occupants and their industry over five centuries. Equinox, Sheffield

[CR75] Hansen AM, Heinrich FBJ (in press) Plant use, foodways, and the agricultural economy of the Ghor as-Safi site complex from the Iron Age to the Mamluk Period: longue durée insights from the macrobotanical assemblage. In: Politis KD (ed) Ancient landscapes of Zoara IIIa: conclusive and specialised studies from surveys and excavations at the Ghor as-Safi in Jordan, 1997–2019. Ionian University, Corfu, pp 169–252

[CR76] Hartman A, Kislev ME (1998) Plant remains from the dwellers of the Ketef Jericho Caves at the end of the Bar-Kokhba revolt. In: Eshel H, Amit D, Porat R (eds) Refuge Caves of the Bar Kokhba revolt. Israel Exploration Society, Tel Aviv, pp 153–168

[CR77] Hartmann-Shenkman A, Kislev ME, Galili E, Melamed Y, Weiss E (2015) Invading a new niche: obligatory weeds at Neolithic Atlit-Yam, Israel. Veget Hist Archaeobot 24:9–18. 10.1007/s00334-014-0498-3

[CR78] Heinrich FBJ (2017) Modelling crop-selection in Roman Italy: the economics of agricultural decision making in a globalizing economy. In: de Haas TCA, Tol GW (eds) The economic integration of Roman Italy: rural communities in a globalizing world. Brill, Leiden, pp 141–169

[CR79] Heinrich FBJ, Hansen AM (2021) A hard row to hoe: ancient climate change from the crop perspective. In: Erdkamp P, Manning JG, Verboven K (eds) Climate change and ancient societies in Europe and the Near East: diversity in collapse and resilience. Palgrave MacMillan, Cham, pp 25–80

[CR80] Heinrich FBJ, Wilkins DA (2014) Beans, boats and archaeobotany: a new translation of *Phasolus*, or why the Romans ate neither kidney beans, nor cowpeas. Palaeohistoria 55/56 (2013/2014):149–176

[CR81] Heiss AG, Stika H-P, De Zorzi N, Jursa M (2013) *Nigella* in the mirror of time: a brief attempt to draw a genus’ ethnohistorical portrait. In: von Carnap-Bornheim C, Dörfler W, Kirleis W, Müller J, Müller U (eds) Von Sylt bis Kastanas: Festschrift für Helmut Johannes Kroll zum 65. Geburtstag. Offa 69/70 (2012/13). Wachholtz, Neumünster, pp 147–169

[CR82] Hopf M (1983) Appendix B. Jericho Plant Remains. In: Kenyon KM, Holland TA (eds) Excavations at Jericho, vol 5: the pottery phases of the Tell and other finds. The British School of Archaeology in Jerusalem, London, pp 576–621

[CR83] Hopf M, Bar-Yosef O (1987) Plant remains from Hayonim Cave. Western Galilee. Paléorient 13:117–120. https://www.jstor.org/stable/41492240

[CR84] Hoppé C (1999) A thousand years of farming: agricultural practices from the Byzantine to Early Ottoman Period at Khirbet Faris, the Kerak Plateau, Jordan. PhD Dissertation. Department of Archaeology and Prehistory, University of Sheffield, Sheffield

[CR85] Hoppé C (2012) Chapter V.15b. The Late Antique Period (Early Byzantine–Umayyad–Early ‘Abbasid): the macroscopic plant remains. In: Politis KD (ed) Sanctuary of Lot at Deir ‘Ain ‘Abata in Jordan. Excavations 1988–2003. Jordan Distribution Agency in association with the British Museum, Amman, pp 518–522

[CR86] Janick J, Hummer KE (2012) The 1500th anniversary (512–2012) of the Juliana Anicia Codex: an illustrated Dioscoridean recension. Chron Hortic 52:9–15

[CR88] Janick J, Stolarczyk J (2012) Ancient Greek illustrated Dioscoridean herbals: origins and impact of the Juliana Anicia Codex and the Codex Neopolitanus. Not Bot Horti Agrobot Cluj-Napoca 40:9–17. 10.15835/nbha4017767

[CR87] Janick J, Paris HS, Parrish DC (2007) The cucurbits of Mediterranean antiquity: identification of taxa from ancient images and descriptions. Ann Bot 100:1441–1457. 10.1093/aob/mcm242 17932073 10.1093/aob/mcm242 PMC2759226

[CR89] Janick J, Whipkey AL, Stolarczyk J (2013) Synteny of images in three illustrated Dioscoridean herbals: Juliana Anicia Codex, Codex Neapolitanus, and Morgan 652. Not Bot Horti Agrobot Cluj-Napoca 41:333–339. 10.15835/nbha4129242

[CR90] Kabukcu C, Hunt C, Hill E, Pomeroy E, Reynolds T, Barker G, Asouti E (2023) Cooking in caves: Palaeolithic carbonised food remains from Franchthi and Shanidar. Antiquity 97:12–28. 10.15184/aqy.2022.143

[CR91] Keimer L (1924) Die Gartenpflanzen Im Alten Ägypten. Ägyptologische studien 1. Hoffmann and Campe, Hamburg

[CR92] Kislev ME (1979) *Triticum parvicoccum* sp. nov., the oldest naked wheat. Isr J Bot 28:95–107. 10.1080/0021213X.1979.10676861

[CR93] Kislev ME (1987) Chalcolithic plant husbandry and ancient vegetation at Shiqmim. In: Levy TE (ed) Shiqmim I: studies concerning Chalcolithic societies in the Northern Negev Desert, Israel (1982–1984). BAR International Series, vol 356. BAR Publishing, Oxford, pp 251–279. (Figures)

[CR94] Kislev ME (1988a) Desiccated plant remains: an interim report. In: Bar-Yosef O, Alon D, Schick T (eds) Naḥal Ḥemar Cave. ‘Atiqot 18. Department of Antiquities and Museums, Jerusalem, pp 76–81

[CR95] Kislev ME (1988b) *Pinus pinea* in agriculture, culture and cult. In: Küster H, Körber-Grohne U (eds) Der prähistorische mensch und Seine umwelt. Festschrift für Udelgard Körber-Grohne zum 65. Geburtstag. Theiss, Stuttgart, pp 73–79

[CR96] Kislev ME (1993) Food remains. In: Finkelstein I, Bunimovich S, Lederman Z (eds) Shiloh. The archaeology of a biblical site. Institute of Archaeology of Tel Aviv University, Tel Aviv, pp 354–361

[CR97] Kislev ME (1997) Early agriculture and paleoecology of Netiv Hagdud. In: Bar-Yosef O, Gopher A (eds) An Early Neolithic village in the Jordan Valley. Part 1: the archaeology of Netiv Hagdud. Harvard University Press, Cambridge, pp 209–236

[CR98] Kislev ME (2009) Reconstructing the ear morphology of ancient small-grain wheat (*Triticum turgidum* ssp. *parvicoccum*). In: Fairbairn A, Weiss E (eds) From foragers to farmers: papers in honour of Gordon C Hillman. Oxbow Books, Oxford, pp 235–238

[CR104] Kislev ME, Simchoni O (2006) Botanical evidence for the arrival of refugees from Judea to Refuge Cave in Nahal Arugot in the fall of 135 CE. Judea Samaria Res Stud (JSRS) 15:141–150. (in Hebrew)

[CR105] Kislev ME, Simchoni O (2009) The secret of the good life at Orhan Mor (Moyat Awad) – a transit station along the incense route. Judea Samaria Res Stud (JSRS) 12:165–176 (in Hebrew)

[CR99] Kislev ME, Artzy M, Marcus E (1993) Import of an Aegean food plant to a Middle Bronze IIA coastal site in Israel. Levant 25:145–154. 10.1179/lev.1993.25.1.145

[CR102] Kislev ME, Hartmann A, Galili E (2004) Archaeobotanical and archaeoentomological evidence from a well at Atlit-Yam indicates colder, more humid climate on the Israeli Coast during the PPNC period. J Archaeol Sci 31:1:301–1310. 10.1016/j.jas.2004.02.010

[CR103] Kislev ME, Melamed Y, Langsam Y (2006a) Plant remains from Tel Batash. In: Panitz-Cohen N, Mazar A (eds) Timnah (Tal Batash) III: the finds from the second millennium BCE. Qedem 45. the Institute of Archaeology. The Hebrew University of Jerusalem, Jerusalem, pp 295–310

[CR101] Kislev ME, Hartmann A, Bar-Yosef O (2006b) Early domesticated fig in the Jordan Valley. Science 312:1(374):372–371. 10.1126/science.1125910 16741119 10.1126/science.1125910

[CR100] Kislev ME, Garfinkel Y, Zohary D (2012) The seeds. In: Garfinkel Y, Dag D, Khalaily H, Marder O, Milevski I, Ronen A (eds) The Pre-Pottery Neolithic B village of Yiftahel: the 1980s and 1990s excavations. Ex Oriente, Berlin, pp 283–286

[CR106] Labuhn I, Finné M, Izdebski A, Roberts N, Woodbridge J (2016) Climatic changes and their impacts in the Mediterranean during the first millennium AD. Late Antiq Archaeol 12:65–88. 10.1163/22134522-12340067

[CR107] LaGro HE (2002) An insight in Ayyubid-Mamluk Pottery: Description and analysis of a corpus of mediaeval pottery from the cane sugar production and village occupation at Tell Abu Sarbut in Jordan. Part I: Text. PhD Dissertation. University of Leiden, Leiden

[CR108] Landa V, Shapira Y, Eliyahu-Behar A, Ben-Arie RL, Weiss E, Reuveni Y, Drori E (2024) Setting the morphologic quality limits enabling accurate classification of charred archaeological grape seeds. Sci Rep 14:16148. 10.1038/s41598-024-66896-z 38997329 10.1038/s41598-024-66896-z PMC11245623

[CR109] Langgut D (2015) Prestigious fruit trees in Ancient Israel: first palynological evidence for growing *Juglans regia* and *Citrus medica*. Isr J Plant Sci 62:98–110. 10.1080/07929978.2014.950067

[CR110] Langgut D (2024) The core area of fruit-tree cultivation: Central Jordan Valley (Levant), ca. 7000 BP. Palynology 48:2347905. 10.1080/01916122.2024.2347905

[CR111] Langgut D, Finkelstein I (2023) Paleo-environment of the Southern Levant during the Bronze and Iron Ages: the pollen evidence. In: Koch I, Lipschits O, Sergi O (eds) From nomadism to monarchy? Revisiting the Early Iron Age Southern Levant. Eisenbrauns, University Park, pp 7–28

[CR112] Langgut D, Garfinkel Y (2022) 7000–year–old evidence of fruit tree cultivation in the Jordan Valley, Israel. Sci Rep 12:7463. 10.1038/s41598-022-10743-6 35523827 10.1038/s41598-022-10743-6 PMC9076912

[CR113] Langgut D, Gleason K, Burrell B (2015) Pollen analysis as evidence for Herod’s Royal garden at the Promontory Palace, Caesarea. Isr J Plant Sci 62:111–121. 10.1080/07929978.2014.975560

[CR114] Langgut D, Shahack-Gross R, Arie E, Namdar D, Amrani A, Le Bailly M, Finkelstein I (2016) Micro-archaeological indicators for identifying ancient cess deposits: an example from Late Bronze Age Megiddo, Israel. J Archaeol Sci Rep 9:375–385. 10.1016/j.jasrep.2016.08.013

[CR115] Langgut D, Tepper Y, Benzaquen M, Erickson-Gini T, Bar-Oz G (2021) Environment and horticulture in the Byzantine Negev Desert, Israel: sustainability, prosperity and enigmatic decline. Quat Int 593–594:160–177. 10.1016/j.quaint.2020.08.056

[CR116] Lentjes D (2016) Landscape and land use in first millennium BC Southeast Italy: planting the seeds of change. Amsterdam University, Amsterdam

[CR117] Lev E (2002) Medicinal substances in Jerusalem from early times to the present day. BAR International Series 1112. Archaeo, Oxford

[CR118] Lev E, Amar Z (2008) Fossils of practical medical knowledge from Medieval Cairo. J Ethnopharmacol 119:24–40. 10.1016/j.jep.2008.05.042 18601991 10.1016/j.jep.2008.05.042

[CR119] Lev-Yadun S, Abbo S (1999) Traditional use of a’kub (*Gundelia tournefortii*, Asteraceae), in Israel and the Palestinian Authority Area. Econ Bot 53:217–219. https://www.jstor.org/stable/4256182

[CR120] Levey M (1966) Medieval Arabic toxicology: the book on poisons of Ibn Wahshīya and its relation to early Indian and Greek texts. Transactions, American Philosophical Society 56. American Philosophical Society, Philadelphia. 10.70249/9798893982879

[CR121] Lightfoot DR (1997) Qanats in the Levant: hydraulic technology at the periphery of early empires. Techno Cult 38:432–451. 10.2307/3107129

[CR122] Lightfoot DR (2000) The origin and diffusion of qanats in Arabia: new evidence from the Northern and Southern Peninsula. Geogr J 166:215–226. http://www.jstor.org/stable/823073

[CR123] Liphschitz N (2000) The archaeobotanical finds. In: Finkelstein I, Ussishkin D, Halpern B (eds) Megiddo III: the 1992–1996 seasons. Emery and Claire Yass Publications in Archaeology, Tel Aviv, pp 487–495

[CR124] Liphschitz N (2020) Wood remains. In: Mazar A, Panitz-Cohen N (eds) Tel Reḥov: A Bronze and Iron Age City in the Beth-Shean Valley. Various objects and Natural-Science Studies, vol 5. Qedem 63. Institute of Archaeology, Hebrew University of Jerusalem, Jerusalem, pp 641–654

[CR125] Liphschitz N, Waisel Y (1992) Part II: Environmental reports, 1: Macrobotanical remains. Section A. In: De Groot A, Ariel DT (eds) Excavations at the city of David 1978-1985: Directed by Yigal Shiloh, Vol 3: Stratigraphical, Environmental, and other Reports. Institute of Archaeology, Hebrew University of Jerusalem, Jerusalem, pp 105–106, 117–12

[CR126] Löw I (1967) Die Flora der Juden, bd. I–IV. Reprografischer nachdruck der Ausgabe Wien und Leipzig (1924–1934). Olms, Hildesheim

[CR127] Mahler-Slasky Y (2004) Philistine material culture as reflected by the archaeobotanical remnants from Ashkelon, Ekron, Gath and Aphek. PhD Dissertation. Bar-Ilan University, Ramat Gan. (in Hebrew with English summary)

[CR128] Mahler-Slasky Y, Kislev ME (2010) *Lathyrus* consumption in Late Bronze and Iron Age sites in Israel: an Aegean affinity. J Archaeol Sci 37:2:477–2485. 10.1016/j.jas.2010.05.008

[CR129] Marzano A (2022) Plants, politics and empire in Ancient Rome. Cambridge University Press, Cambridge

[CR130] Mascher M, Schuenemann VJ, Davidovich U et al (2016) Genomic analysis of 6,000-year-old cultivated grain illuminates the domestication history of barley. Nat Genet 48:1:089–1093. 10.1038/ng.3611 10.1038/ng.3611 27428749

[CR131] Mazzaoui MF (1981) The Italian cotton industry in the Later Middle Ages 1100–1600. Cambridge University Press, Cambridge

[CR132] Meiri M, Bar-Oz G (2024) Unraveling the diversity and cultural heritage of fruit crops through paleogenomics. Trends Genet 40:398–409. 10.1016/j.tig.2024.02.003 38423916 10.1016/j.tig.2024.02.003 PMC11079635

[CR133] Melamed Y, Kislev ME (2005) Remains of seeds, fruits and insects from the excavations of the village of ‘Ein Gedi. ‘Atiqot 49:89–102. (in Hebrew)

[CR134] Melamed Y, Plitmann U, Kislev ME (2008) *Vicia peregrina*: an edible Early Neolithic legume. Veget Hist Archaeobot 17(Suppl1):29–34. 10.1007/s00334-008-0166-6

[CR135] Mikhail MSA (2000) Some observations concerning edibles in Late Antique and Early Islamic Egypt. Byzantion 70:105–121. https://www.jstor.org/stable/44172365

[CR136] Mir-Makhamad B, Spengler RN III (2023) Testing the applicability of Watson’s Green Revolution concept in first millennium CE Central Asia. Veget Hist Archaeobot. 10.1007/s00334-023-00924-2 10.1007/s00334-023-00924-2 PMC12881048 41659818

[CR137] Nasrallah N (2010) Annals of the caliphs’ kitchens. Ibn Sayyār al-Warrāq’s Tenth-Century Baghdadi cookbook. Brill, Leiden

[CR138] Neef R (1989) Plants. In: van der Kooij G, Ibrahim MM (eds) Picking up the threads: a continuing review of excavations at Deir Alla, Jordan. University of Leiden, Archaeological Centre, Leiden, pp 30–37

[CR139] Orendi A, Yehuda E, Zeischka-Kenzler A, Tal O (2023) Flora in the Latin East: archaeobotanical remains from Crusader Arsur. Tel Aviv 50:263–288. 10.1080/03344355.2023.2246822

[CR140] Ortloff CR (2020) Hydraulic engineering at 100 BC–AD 300 Nabataean Petra (Jordan). Water 12:3498. 10.3390/w12123498

[CR141] Paris HS (2015) Origin and emergence of the sweet dessert watermelon, *Citrullus lanatus*. Ann Bot 116:133–148. 10.1093/aob/mcv077 26141130 10.1093/aob/mcv077 PMC4512189

[CR142] Paris HS (2023) Origin of the dessert watermelon. In: Dutta SK, Nimmakayala P, Reddy UK (eds) The watermelon genome. Compendium of plant genome. Springer, Cambridge, pp 1–16. 10.1007/978-3-031-34716-0_1

[CR143] Paris HS, Daunay M-C, Janick J (2012) Occidental diffusion of cucumber (*Cucumis sativus*) 500–1300 CE: two route to Europe. Ann Bot 109:117–126. https://www.jstor.org/stable/43576608 22104164 10.1093/aob/mcr281 PMC3241595

[CR144] Politis KD (2013a) The sugar industry in the Ghawr aṣ-Ṣāfī, Jordan. Stud Hist Archaeol Jordan 11:467–480

[CR145] Politis KD (2013b) Agriculture in the Ghor es-Safi during the Byzantine and Islamic periods. In: Bakhit MA, al-Kahwati HM (eds) Agriculture in Bilād al-Shām from Late Byzantine times to the end of the Ottoman Period. The 9th International conference on the history of Bilād al-Shām 10–14 Jumada I 1433 A.H. / 1–5 April 2012, vol 3. University of Jordan Press, Amman, pp 697–717

[CR146] Politis KD (2015) Earliest evidence for an Arab sugarcane industry with a focus on the southern Bilad ash-Sham. In: Politis KD (ed) The origins of the sugar industry and the transmission of Ancient Greek and Medieval Arab science and technology from the Near East to Europe. Proceedings of the International Conference, Athens 23 May 2015. National and Kapodistriako University of Athens, Athens, pp 25–42

[CR147] Politis KD (2021) Historical background. In: Politis KD (ed) Ancient landscapes of Zoara I: surveys and excavations at the Ghor as-Safi in Jordan, 1997–2018. Routledge, Abingdon, pp 20–29

[CR148] Rambeau CMC (2010) Palaeoenvironmental reconstruction in the Southern Levant: synthesis, challenges, recent developments and perspectives. Philos Trans R Soc A 368:5225–5248. 10.1098/rsta.2010.0190 10.1098/rsta.2010.0190 20956369

[CR149] Ramos-Madrigal J, Wiborg Runge AK, Bouby L et al (2019) Palaeogenomic insights into the origins of French grapevine diversity. Nat Plants 5:595–603. 10.1038/s41477-019-0437-5 31182840 10.1038/s41477-019-0437-5

[CR150] Ramsay J (2010) Trade or trash: an examination of the archaeobotanical remains from the Byzantine harbour at Caesarea Maritima, Israel. Int J Naut Archaeol 39:376–382. 10.1111/j.1095-9270.2010.00267.x

[CR151] Ramsay J (2013) 10.D Plant remains. In: Oleson JP, Schick R (eds) Humayma excavation project, 2: Nabataean campground and necropolis, Byzantine churches, and Early Islamic domestic structures. American Schools of Oriental Research, Boston, pp 351–358

[CR152] Ramsay J, Mueller N (2016) Telling seeds: archaeobotanical investigations at Tall al-‘Umayri, Jordan. In: McGeough KM (ed) The archaeology of agro-pastoralist economies in Jordan. American Schools of Oriental Research, Boston, pp 1–25

[CR153] Ramsay JH, Parker ST (2016) A diachronic look at the agricultural economy at the Red Sea Port of Aila: an archaeobotanical case for hinterland production in arid environments. Bull Am Schools Orient Res (BASOR) 376:101–120. 10.5615/bullamerschoorie.376.0101

[CR154] Ramsay J, Smith AM (2013) Desert agriculture at Bir Madhkur: the first archaeobotanical evidence to support the timing and scale of agriculture during the Late Roman/Byzantine Period in the hinterland of Petra. J Arid Environ 99:51–63. 10.1016/j.jaridenv.2013.09.005

[CR155] Ramsay J, Tepper Y, Weinstein-Evron M, Aharonovich S, Liphschitz N, Marom N, Guy Bar-Oz G (2016) For the birds – an environmental archaeological analysis of Byzantine pigeon towers at Shivta (Negev Desert, Israel). J Archaeol Sci Rep 9:718–727. 10.1016/j.jasrep.2016.08.009

[CR156] Riehl S (2000) Erste ergebnisse der archäobotanischen untersuchungen am Tall Mozan/Urkeš. Mitteilungen der Deutschen Orient-Gesellschaft (MDOG) 132:229–238

[CR157] Riehl S (2004) Archaeobotany at the Early Bronze Age settlement of Ḫirbet ez-Zeraqōn: a preliminary report. Z Des Deutschen Palästina-Vereins (ZDPV) 120:101–122. https://www.jstor.org/stable/27931744

[CR158] Riehl S (2010) Plant production in a changing environment: the archaeobotanical remains from Tell Mozan. In: Deckers K, Doll M, Pfälzner P, Riehl S (eds) The development of the environment, subsistence and settlement of the City of Urkeš and its region. Harrassowitz, Wiesbaden, pp 13–158

[CR159] Rosen SA (2017) Basic instabilities? Climate and culture in the Negev over the long term. Geoarchaeology 32:6–22. 10.1002/gea.21572

[CR160] Roskin J, Taxel I (2021) He who revives dead land: groundwater harvesting agroecosystems in sand along the southeastern Mediterranean Coast since Early Medieval times. Mediterr Geosci Rev 3:293–318. 10.1007/s42990-021-00058-5

[CR161] Samuel D (2001) Archaeobotanical evidence and analysis. In: Berthier S (ed) Peuplement rural et aménagements hydroagricoles dans la moyenne vallée de l’Euphrate fin VIIe–XIXe siècle. Institut Français d’Études Arabes de Damas, Damascus, pp 343–481

[CR162] Sato T (2004) Sugar in the economic life of Mamluk Egypt. Mamlūk Stud Rev (MSR) 8:87–107. 10.6082/M15T3HM2

[CR163] Sato T (2015) Sugar in the social life of Medieval Islam. Brill, Leiden

[CR164] Shamir O, Hildebrandt B, Galili R, Shamir N, Bar-Oz G (2023) Trash or cache? The textile evidence from the Naḥal ‘Omer middens as indicator of Early-Islamic period trade networks along Israel’s silk road. ‘Atiqot 112:175–200

[CR165] Sheffer A, Tidhar A (1991) The textiles from the ‘En-Boqeq excavation in Israel. Text Hist 22:3–46. 10.1179/004049691793711405

[CR166] Shepperd WD (2008) *Ceratonia siliqua* L. carob. In: Bonner FT, Karrfalt RP (eds) The Woody plant seed manual. Agriculture handbook 727. USDA Forest Service, Washington, DC, pp 371–373

[CR167] Simchoni O, Kislev ME, Melamed Y (2007) Botanical remains. Chapter 15A: Beth-Shean as a trade center for crops in the Bronze Age: botanical and entomological evidence. In: Mazar A, Mullins RA (eds) Excavations at Tel Beth-Shean 1989–1996. The Middle and Late Bronze Age Strata in Area, vol 2. The Israel Exploration Society, Jerusalem, pp 702–715

[CR168] Spengler RN III, Stark S, Zhou X et al (2021) A journey to the West: the ancient dispersal of rice out of East Asia. Rice 14:83. 10.1186/s12284-021-00518-4 34564763 10.1186/s12284-021-00518-4 PMC8464642

[CR169] Steiner ML (2008) Tell Abu Sarbut: the occupation of a rural site in the Ayyubid / Mamluk periods. In: Steiner ML, van der Steen EJ (eds) Sacred and sweet: studies on the material culture of tell Deir ‘Alla and Tell Abu Sarbut. Peeters, Leuven, pp 157–196

[CR170] Stern EJ (1999) The Sugar Industry in Palestine during the Crusader, Ayyubid and Mamluk Periods in Light of the Archaeological Finds. Unpublished M.A. Dissertation. The Hebrew University, Jerusalem

[CR171] Stern EJ, Getzov N, Shapiro A, Smithline H (2015) Sugar Production in the ‘Akko Plain from the Fatimid to the Early Ottoman Periods. In: Politis KD (ed) The origins of the sugar industry and the transmission of Ancient Greek and Medieval Arab science and technology from the Near East to Europe. Proceedings of the International Conference, Athens 23 May 2015. National and Kapodistriako University of Athens, Athens, pp 79–112

[CR172] Stewart A (1887) Of the holy places visited by Antoninus Martyr (circ. 560–570 A.D.). Palestine Pilgrims’ Text Society, London

[CR173] Stiebel GD (2018) Burns like Fire – Mustard and viniculture in Roman Palestine. Tarbiẕ 86:5–37 (in Hebrew). http://www.jstor.org/stable/26828774

[CR174] Tabak Y (2006) Agricultural prosperity in Roman Israel confirmed by archeobotanical finds. MSc Dissertation. Bar-Ilan University, Ramat Gan. (in Hebrew)

[CR175] Taha H (2004) Die ausgrabungen von Ṭawāḥīn es-Sukkar Im Jordan-Tal. Z Dtsch Paläst-Ver 120:73–78. https://www.jstor.org/stable/27931734

[CR176] Taha H (2009) Some aspects of sugar production in Jericho, Jordan Valley. In: Kaptijn E, Petit LP (eds) A timeless vale: archaeological and related essays on the Jordan Valley in honour of Gerrit Van der Kooij on the occasion of his sixty-fifth birthday. Leiden University, Leiden, pp 181–191

[CR177] Taha H (2015) The sugarcane industry in Jericho, Jordan Valley. In: Politis KD (ed) The origins of the sugar industry and the transmission of Ancient Greek and Medieval Arab science and technology from the Near East to Europe: Proceedings of the International Conference, Athens 23 May 2015. National and Kapodistriako University of Athens, Athens, pp 51–77

[CR178] Taxel I, Roskin J (2025) Early Islamic groundwater-harvesting plot-and-berm agroecosystems along the Southeastern Mediterranean Coast: the earliest known agriculture in sand. Environ Archaeol. 10.1080/14614103.2025.2452090

[CR179] Touchan R, Meko D, Hughes MK (1999) A 396-year reconstruction of precipitation in Southern Jordan. J Am Water Resour Assoc 35:49–59

[CR180] Valera J, Matilla-Seiquer G, Obón C, Rivera D (2022) Archaeobotanical study of Tell Khamîs (Syria). Heritage 5:1687–1718. 10.3390/heritage5030088

[CR181] Van der Veen M (2010) Agricultural innovation: invention and adoption or change and adaptation? World Archaeol 42:1–12. 10.1080/00438240903429649

[CR182] Van der Veen M (2011) Consumption, trade and innovation: exploring the botanical remains from the Roman and Islamic ports at Quseir al-Qadim, Egypt. Africa Magna, Frankfurt am Main

[CR183] Van Zeist W, Heeres JAH (1973) Paleobotanical studies of Deir ‘Alla. Jordan Paléorient 1:21–37. https://www.jstor.org/stable/41489695

[CR184] Varisco D, Fuks D (2024) In memoriam: Andrew Watson’s contribution to the history of agriculture: the Islamic Green Revolution Fifty years on. J Econ Hist. https://www.cambridge.org/core/journals/journal-of-economic-history/announcements/in-memoriam

[CR186] Walker BJ (2004) Mamluk investment in Transjordan: a boom and bust economy. Mamlūk Stud Rev (MSR) 8:119–147. 10.6082/M1222RX3

[CR187] Walker BJ (2011) Jordan in the Late Middle Ages: transformation of the Mamluk frontier. Middle East Documentation Center, Chicago

[CR185] Wallace M, Bonhomme V, Russell J et al (2019) Searching for the origins of Bere barley: a geometric morphometric approach to cereal landrace recognition in archaeology. J Archaeol Method Theory 26:1:125–1142. 10.1007/s10816-018-9402-2

[CR188] Watson AM (1974) The Arab Agricultural Revolution and its diffusion. J Econ Hist 34:700–1100. 10.1017/S0022050700079602

[CR189] Watson AM (1981) A Medieval Green Revolution: new crops and farming techniques in the Early Islamic world. In: Udovitch AL (ed) The Islamic Middle East, 700 – 1900: studies in economic and social history. Darwin, Princeton, pp 29–58

[CR190] Watson AM (1983) Agricultural innovation in the Early Islamic world: the diffusion of crops and farming techniques. Cambridge University Press, Cambridge, pp 700–1100

[CR191] Weiss E (2015) Beginnings of fruit growing in the Old World – two generations later. Isr J Plant Sci 62:75–85. 10.1080/07929978.2015.1007718

[CR193] Weiss E, Zohary D (2011) The Neolithic Southwest Asian founder crops. Their biology and archaeobotany. Curr Anthropol 52(Suppl4):5237–5254. 10.1086/658367

[CR192] Weiss E, Mahler-Slasky Y, Melamed Y, Lederman Z, Bunimovitz S, Bubel S, Manor D (2019) Foreign food plants as prestigious gifts: the archaeobotany of the Amarna Age palace at Tel Beth-Shemesh, Israel. Bull Am Sch Orient Res (BASOR) 381:83–105. 10.1086/703342

[CR194] White CE, Makarewicz CA (2012) Harvesting practices and Early Neolithic barley cultivation at el-Hemmeh, Jordan. Veget Hist Archaeobot 21:85–94. 10.1007/s00334-011-0309-z

[CR195] Whitlam J, Finlayson B, Bogaard A, Charles M, Makarewicz CA (2023) Processing and storage of tree fruits, cereals and pulses at PPNA Sharara, southern Jordan. Veget Hist Archaeobot 32:501–516. 10.1007/s00334-023-00938-w

[CR196] Wright CA (2009) Did the ancients know the artichoke? Gastronomica 9:21–28. 10.1525/gfc.2009.9.4.21

[CR197] Zaitschek DV (1961) Remains of cultivated plants from the caves of Naḥal Mishmar: preliminary note. Isr Explor J 11:70–72. https://www.jstor.org/stable/27924845

[CR198] Zaitschek DV (1980) Appendix A. Plant remains from the Cave of the Treasure. In: Bar-Adon P (ed) The Cave of the Treasure: the finds from the caves in Nahal Mishmar. Israel Exploration Society, Jerusalem, pp 223–228

[CR199] Zech-Matterne V, Tengberg M, van Andringa W (2015) *Sesamum indicum* L. (sesame) in 2nd century BC Pompeii, Southwest Italy, and a review of early sesame finds in Asia and Europe. Veget Hist Archaeobot 24:673–681. 10.1007/s00334-015-0521-3

[CR200] Zohary M (1966) Flora Palaestina. Part One: Equisetaceae to Moringaceae, 2 vols: text and plates. The Israel Academy of Sciences and Humanities, Jerusalem

[CR201] Zohary M (1972) Flora Palaestina. Part Two. Platanaceae to Umbelliferae, 2 vols: text and plates. The Israel Academy of Sciences and Humanities, Jerusalem

[CR202] Zohary D (2002) Domestication of the Carob (*Ceratonia siliqua* L). Isr J Plant Sci 50(Supp1):141–145. 10.1560/BW6B-4M9P-U2UA-C6NN

[CR203] Zohary D, Hopf M, Weiss E (2012) Domestication of plants in the Old World: the origin and spread of domesticated plants in Southwest Asia, Europe, and the Mediterranean Basin, 4th edn. Oxford University Press, Oxford

